# HSL1 and BAM1/2 impact epidermal cell development by sensing distinct signaling peptides

**DOI:** 10.1038/s41467-022-28558-4

**Published:** 2022-02-15

**Authors:** Andra-Octavia Roman, Pedro Jimenez-Sandoval, Sebastian Augustin, Caroline Broyart, Ludwig A. Hothorn, Julia Santiago

**Affiliations:** 1 The Plant Signaling Mechanisms Laboratory, Department of Plant Molecular Biology, University of Lausanne, 1015 Lausanne, Switzerland; 2 Institute of Biostatistics, Leibniz University, 30167 Hannover, Germany

**Keywords:** Plant signalling, X-ray crystallography

## Abstract

The membrane receptor kinases HAESA and HSL2 recognize a family of IDA/IDL signaling peptides to control cell separation processes in different plant organs. The homologous HSL1 has been reported to regulate epidermal cell patterning by interacting with a different class of signaling peptides from the CLE family. Here we demonstrate that HSL1 binds IDA/IDL peptides with high, and CLE peptides with lower affinity, respectively. Ligand sensing capability and receptor activation of HSL1 require a SERK co-receptor kinase. Crystal structures with IDA/IDLs or with CLE9 reveal that HSL1-SERK1 complex recognizes the entire IDA/IDL signaling peptide, while only parts of CLE9 are bound to the receptor. In contrast, the receptor kinase BAM1 interacts with the entire CLE9 peptide with high affinity and specificity. Furthermore, the receptor tandem BAM1/BAM2 regulates epidermal cell division homeostasis. Consequently, HSL1-IDLs and BAM1/BAM2-CLEs independently regulate cell patterning in the leaf epidermal tissue.

## Abstract (web summary)

HAESA and HSL2 are receptors for IDA/IDL-family signaling peptides in plants. Here the authors show that HSL1, previously shown to recognize CLE9, preferentially binds IDA/IDL peptides and regulates leaf epidermal patterning independently of CLE peptides.

## Introduction

Plant peptide hormones have emerged as key players in cell-to-cell communication. They coordinate plant immunity, development, and adaptations to an ever-changing environment^1^. To sense sequence-diverse signaling molecules, plants have evolved unique membrane receptor kinases. Leucine-Rich Repeat Receptor Kinases (LRR-RKs) represent the largest class of membrane receptor kinases in plants^2^. Their cognate peptide ligands are processed from larger preproteins and can be post-translationally modified^1,3^, for example by tyrosine sulfation, proline hydroxylation (Hyp), and hydroxyproline arabinosylation. These modifications may regulate the diffusion, stability and bioactivity of the peptide hormone, by for example allowing for the specific recognition by their cognate LRR-RK^4–8^.

INFLORESCENCE DEFICIENT IN ABSCISSION (IDA) and IDA-LIKE (IDL) are a family of dodeca hydroxyprolinated peptides that are sensed by the LRR-RKs HAESA (HAE) and HAESA-LIKE 2 (HSL2) involved in regulating cell separation events, such as organ shedding and root cap sloughing^4,9–12^. SOMATIC EMBRYOGENESIS RECEPTOR KINASE (SERK) proteins act as co-receptors mediating high-affinity ligand sensing and receptor complex activation^4,13,14^. In the HAE-IDA-SERK1 complex structure, a central hydroxyproline anchors the IDA peptide to the peptide-binding pocket in HAE^4^. IDA/IDL peptides are sequence related to the well-studied CLAVATA3/EMBRYO SURROUNDING REGION-RELATED (CLE) peptide family consisting of 27 members^15^. CLEs also undergo post-translational modifications such as proline hydroxylation and arabinosylation^8,16–18^ that regulate peptide bioactivity^19^. CLE peptides have been implicated in shoot stem cell homeostasis, root xylem development, root protophloem differentiation, vascular cambium activity, and stomata formation^8,16,20–22^, and are recognized by different LRR-RKs^6,17,21,23–26^. The LRR-RK BARELY ANY MERISTEM 1 (BAM1) and its homolog BAM2 sense CLV3^27^ and other CLE peptides with nanomolar affinities^6,26^, and coordinate formative root divisions, male gametophyte development, root xylem patterning and meristems development^21,26–28^. CLAVATA3 INSENSITIVE RECEPTOR KINASES (CIKs)^29^ have been genetically described as the co-receptors for CLV1 and BAMs. Recently, CLE9 has been proposed to act as a dual ligand for the LRR-RKs BAM1 and HAESA-LIKE 1 (HSL1), to regulate root xylem file number and stomatal cell lineage development, respectively^21,22^. With respect to its role in stomatal development, the CLE9 sensing system has been reported to operate independently from the well-characterized ERECTA (ER) - EPIDERMAL PATTERNING FACTORs (EPFs) - SERK signaling pathway^21,30–35^, suggesting the existence of additional regulatory layers in the control of epidermal cell development.

Following up on our previous work on HAE-IDA/IDL-SERK interaction, we characterize how the related HSL1 receptor can discriminate between IDA/IDLs and the sequence-related CLE peptide family members. Here we report that HSL1 is a bona-fide receptor for IDA/IDL peptides that have evolved a specific binding surface for high-affinity recognition of all IDA/IDL family members. In contrast, the sequence-related CLE9 peptide is sensed by the LRR-RK BAM1 with high affinity and specificity. At the physiological level, we find that HSL1-IDLs and BAM1/BAM2-CLEs control different aspects of leaf epidermal cell patterning. Structural analysis of HSL1-IDL/CLE9 and BAM1-CLE9 complexes reveals how two families of LRR-RKs have evolved to specifically sense two distinct, yet sequence-related, signaling peptide families.

## Results

**Fig. 1:**
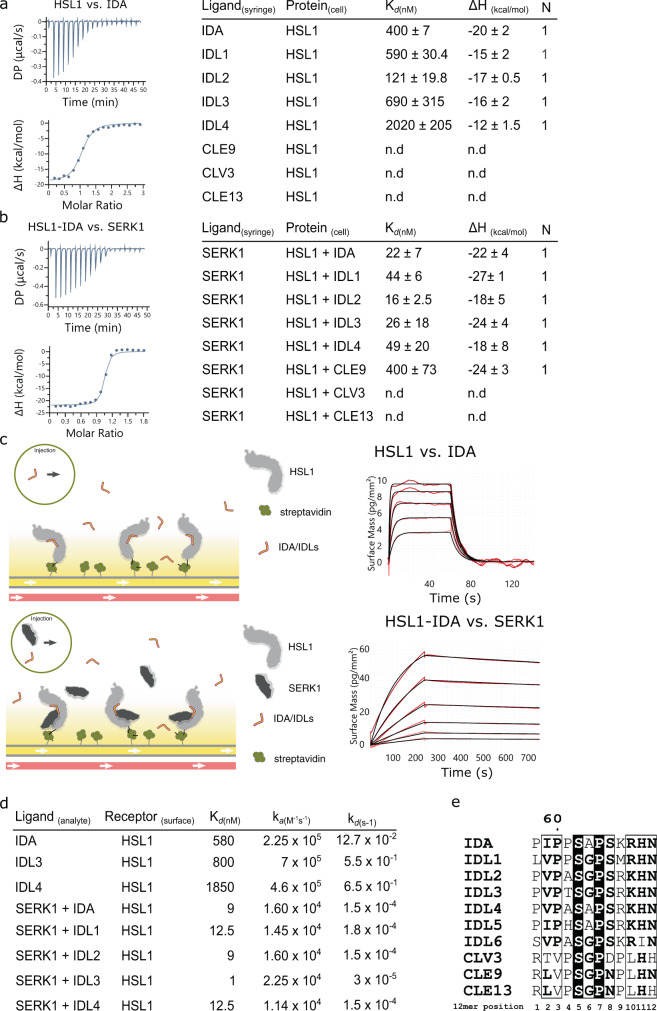
HSL1 senses IDA/IDL peptides with high affinity and forms a stable complex with the co-receptor SERK1. **a** ITC binding experiments and summary table of IDA/IDL and CLE peptides versus HSL1 ectodomain. *K*_d_ (dissociation constant) indicates the binding affinity between the two molecules considered (in nanomolar). The N indicates the reaction stoichiometry (*n* = 1 for a 1:1 interaction). The values indicated in the table are the mean ± SD of at least two independent experiments. **b** Contribution of the SERK1 co-receptor to the HLS1-IDA/IDLs and HSL1-CLE ternary complex formation. ITC experiments and results table of titrating SERK1 protein into a solution containing HSL1 and the indicated peptide. **c** Schematic overview of the GCI binding experiments. Experiments were done using Avi-tag-based coupling. Streptavidin (in green) was immobilized using direct amine coupling to the chip. Next, the biotinylated ectodomain of HSL1 was captured by streptavidin. IDA/IDLs (top) or SERK1 + IDA/IDLs (bottom) were used as analytes in the different experiments. Binding kinetics of HSL1 receptor vs. IDA, and HSL1-IDA complex vs. SERK1 obtained from GCI experiments. The sensograms with recorded data are shown in red with the respective fits in black. **d** GCI summary table of HSL1 vs. IDA/IDLs and contribution of SERK1 to the kinetics of the ternary complex formation. The table contains the corresponding association rate constant (*k*_a_), dissociation rate constant (*k*_d_), and the dissociation constant *K*_d_ from experiments reported in Supplementary Fig. 4. **e** Sequence alignment of the mature peptides of IDA/IDLs, and CLE9, CLV3, and CLE13.

It has been previously reported that the LRR-RKs HAE and HSL2 specifically bind IDA and other IDL peptides to regulate abscission and cell separation processes^4,10–12^. To characterize the sequence-related HSL1 receptor, we determined the crystal structure of the apo HSL1 ectodomain at 1.83 Å resolution (Supplementary Fig. 1 and Supplementary Table 1). HSL1 folds into a superhelical assembly of 21 LRRs with a conserved ligand-binding surface (Supplementary Fig. 1), suggesting a shared peptide recognition mechanism with the receptors HAE/HSL2. We next investigated whether HSL1 can sense IDA/IDL peptides and form a ternary complex with the co-receptor protein SERK1^4,14^. HSL1 forms stable heterodimeric complexes with SERK1 in the presence of IDA/IDL peptides in analytical size-exclusion chromatography experiments (SEC) (Supplementary Fig. 1). To quantify these interactions, we titrated IDA and IDL peptides into a solution containing isolated HSL1 ectodomain, using isothermal titration calorimetry (ITC). We found that IDA and IDL1-4 bind to the HSL1 receptor-ligand pocket with high affinity, ranging from 120 nM to 2 µM (Fig. 1a and Supplementary Fig. 2). We next assessed the contribution of SERK1 to IDA/IDLs recognition by HSL1. Our calorimetry experiments reveal that SERK1 binds HSL1 in the presence of IDA/IDL peptides with a dissociation constant (*K*_d_) of around 15 to 50 nM, indicating that IDA/IDLs promote a high-affinity association between HSL1 and the co-receptor SERK1 (Fig. 1b and Supplementary Fig. 3). In contrast, we did not detect binding of CLE9 to the isolated ectodomain of HSL1 in ITC assays. Similarly, we could not detect binding with other CLE family members such as CLV3 or the CLE9 sequence-related CLE13 (Fig. 1a and Supplementary Fig. 2). In the presence of the co-receptor SERK1, we did observe CLE9 binding to HSL1 with a dissociation constant of 400 nM. In the case of CLV3 and CLE13 no biding was detected in the presence of SERK1 (Fig. 1b and Supplementary Fig. 3).

We next determined binding kinetic parameters for HSL1 vs. IDA peptides and the co-receptor SERK1 in grating-coupled interferometry (GCI) assays^36,37^. GCI experiments yielded similar *K*_d_s between HSL1 and IDA peptides, and with the SERK co-receptor (Fig. 1c-d and Supplementary Fig. 4). The interaction between the co-receptor and HSL1 in the presence of the IDA ligands is defined by a fast association (*k*_*a*_ ∼ 1.6 × 10^4 ^M^−1^ s^−1^) and a very slow dissociation rate (*k*_d_ ∼ 1.5 × 10^−4 ^s^−1^) (Fig. 1d and Supplementary Fig. 4). Taken together, our quantitative binding experiments demonstrate that HSL1 is a bona-fide IDA/IDLs receptor and uses SERK proteins as co-receptor kinases for high-affinity ligand sensing.

The vastly different binding affinities between IDA/IDLs vs. CLE9 to HSL1 prompted us to determine co-crystal structures of four HSL1-IDA/IDL-SERK1 ternary complexes with: IDA, IDL1, IDL2, and IDL3 (at 2.31 Å, 2.38 Å, 2.20 Å, and 2.70 Å, respectively) and the HSL1-CLE9-SERK1 complex at 2.87 Å (Supplementary Fig. 5 and Supplementary Table 1). We first validated our HSL1-IDA-SERK1 structural model, by mutating key amino acids in the HSL1 ligand-binding surface and its interface with SERK1. Size-exclusion chromatography experiments of HSL1 mutants: HSL1^Y261AR402A^ (ligand pocket) and HSL1^N543AY544AN566A^ (receptor-co-receptor zipper-like interface), disrupted the formation of the ternary complex (Supplementary Fig. 6). The corresponding mutations in HAE (HAE^F268AR409A^ and HAE^N550AY551AN573A^) fail to form stable signaling complexes and did not restore floral abscission in the *hae hsl2* mutant background (Supplementary Fig. 6 and Supplementary Fig. 7), suggesting that HSL1 shares a common peptide recognition mechanism with other HAE-type receptors.

**Fig. 2:**
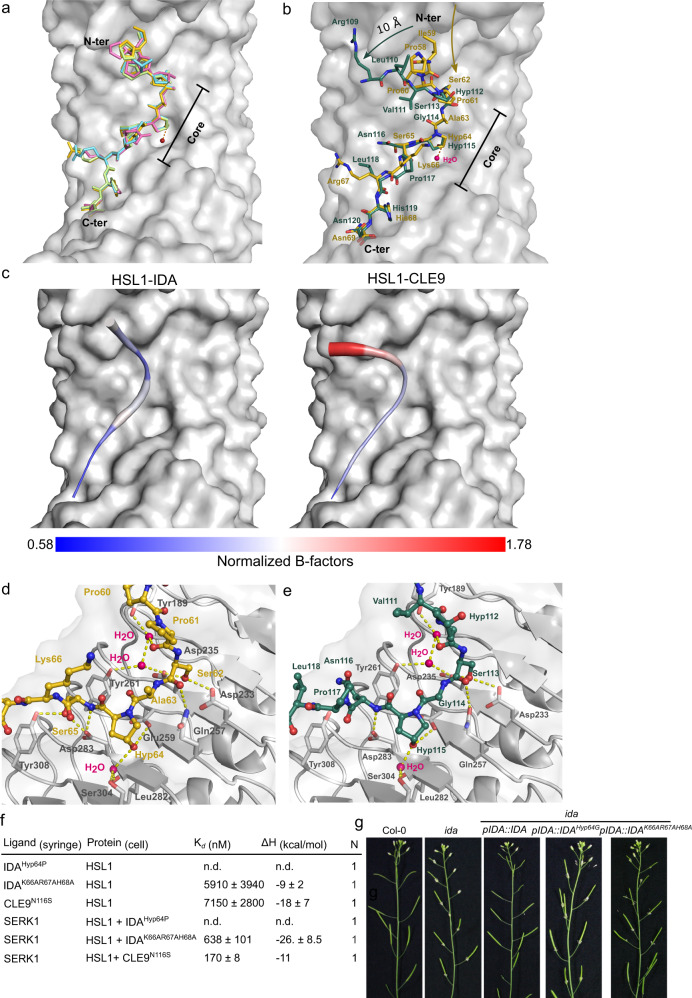
HSL1 discriminates between IDA/IDLs and the CLE9 peptide via the recognition of specific structural features in the N-terminal and core peptide regions. **a** IDA/IDL peptides bind to the HSL1 binding groove in a fully extended conformation. Structural superimposition of IDA (yellow bond representation), IDL1 (blue), IDL2 (green), and IDL3 (pink) in the HSL1 binding canyon (light gray). The conserved central hydroxyproline is depicted together with the crystallographic water molecule that connects it to the receptor. **b** CLE9 falls out of the HSL1 binding pocket. Close view of the structural superimposition of CLE9 (green) and IDA (yellow) in bond representation bound to the HSL1 ligand groove. The yellow arrow indicates the direction of the natural binding canyon followed by IDA (yellow), whereas the green arrow highlights the displacement (10 Å) of the N-terminal of CLE9 (green) with respect to the HSL1 binding surface. **c** Temperature factor analysis of IDA and CLE9 peptides bound to HSL1 reveals a high atomic displacement parameter for the N-terminal region of CLE9 compared to IDA. Peptides are colored according to the scale of normalized B-factor values (ranging from 0.58 to 1.78, bottom). Normalization was made using the Cα B-factor of the complexes as follows Bi’= Bi/ √(Σ Bi^2^/*n*). The surface of the HSL1 receptor is depicted in gray. **d** The core region of IDA/IDL peptides is specifically recognized by the receptor HSL1 through a highly coordinated network of hydrogen bonds. **e** CLE9 is missing a key Ser residue in position Asn116 for anchoring the peptide to the HSL1 receptor. Close up view of the core region of CLE9 (green bond representation) in complex with HSL1 (light gray). Hydrogen bonds are depicted in yellow, and waters are shown as bright pink spheres. **f** Binding analysis of IDA^Hyp64P^, IDA^K66AR67AH68A^, and CLE9^N116S^ mutants vs. HSLI and SERK1. ITC table summaries. *K*_d_ dissociation constant, N stoichiometry (*n* = 1 for a 1:1 interaction). The values indicated in the table are the mean ± SD of at least two independent experiments. **g** IDA requires the Hyp64 and its C-terminal region for bioactivity. Abscission complementation assays of *ida* mutant with wild-type IDA and the IDA variants Pro64 to Gly and the triple mutant Lys66, Arg67, and H68A to Ala, driven by its endogenous promoter. A minimum of two independent lines were analyzed for floral abscission phenotype.

In agreement with our biochemical assays, the crystal structures revealed that all IDA/IDL peptides specifically bind to the peptide-binding surface of HSL1. IDA/IDLs adopt a fully extended conformation in the structure (Fig. 2a and Supplementary Fig. 5). The co-receptor SERK1 interacts with the C-terminal region of the different IDA/IDL peptides (residues 68-69 in IDA), completing the peptide-binding pocket using its N-terminal loop region (Supplementary Fig. 6). Superposition of the four crystal structures yielded an R.M.S.D of ~0.6 Å comparing 590 Cα atom pairs, with IDL peptides perfectly aligning to each other (Fig. 2a). Given the high structural similarity among all IDA/IDL ternary complexes, we subsequently used the HSL1-IDA-SERK1 complex to further analyze the distinct ligand recognition mechanism between the IDA/IDLs and CLE peptide families.

Structural superposition of the HSL1-IDA-SERK1 and HSL1-CLE9-SERK1 complexes revealed that both IDA and CLE9 bind to the HSL1 peptide-binding surface, however, with the CLE9 N-terminus (residues 109-112) adopting a radically different orientation compared to IDA and IDLs (Fig. 2b and Supplementary Fig. 5). While the proline-rich N-terminus of IDA/IDLs accommodate into the hydrophobic cavity of the HSL1 binding surface (Supplementary Fig. 6), the corresponding region of CLE9 is unable to bind and found shifted 10 Å away from the binding groove in our structure (Fig. 2b). The lack of receptor-peptide interaction in this region is supported by the poorly defined electron density and the high atomic displacement parameters (B-factors) that indicate high flexibility of the CLE9 N-terminus (Fig. 2c and Supplementary Fig. 5). Taken together, HSL1 can sense the full mature IDA/IDL peptides but not CLE9, with major structural differences found in the N-terminus of the two peptide families.

We next analyzed the structural differences in the peptides core motifs. The IDA core motif contains three conserved amino acids (Ser62^IDA^, Hyp64^IDA^, and Ser65^IDA^) that are specifically recognized by the receptor through a network of direct and water-mediated hydrogen bonds that anchor the peptide to the receptor (Fig. 2d and Supplementary Fig. 5). Removal of the hydroxyl group of the central Pro64 in IDA results in the loss of binding to HSL1 and disrupts the formation of the ternary complex (Fig. 2f and Supplementary Fig. 8)^4^. In line with this, plants expressing the IDA^Hyp64Gly^ mutant protein under the expression of the IDA promoter failed to rescue the abscission defect of the loss-of-function *ida* mutant, in contrast to the wild-type IDA (Fig. 2g and Supplementary Fig. 6). These results highlight the relevance of the peptide core binding region in IDA bioactivity.

CLE9 and IDA/IDL peptides share sequence similarities in their core regions such as the Ser in position five (Ser113 in CLE9) and the Hyp in position 7 (Hyp115 in CLE9)^17^, enabling CLE9 to bind HSL1–SERK1 with lower affinity (Fig. 1a, b). However, Ser65^IDA^, which makes direct contacts with Asp283 and Tyr308 in HSL1 (Fig. 2d), is an Asn residue in CLE9 that is unable to interact with the receptor-binding surface (Fig. 2e). Replacing Asn116^CLE9^ to a Ser results in a 2-fold increase in binding affinity to the HSL1–SERK1 complex and to detectable binding to the isolated HSL1 ectodomain (Fig. 2f and Supplementary Fig. 8).

The C-terminus of IDA/IDL and CLE9 are similar, with the last two residues being conserved (Fig. 1e). This motif interacts with the HSL1 receptor and with the SERK1 N-terminal loop^4^. In this region, receptor-peptide and peptide-co-receptor interactions are mainly mediated by backbone interactions. His68^IDA^ acts as a bridging point between HSL1 and the SERK1 co-receptor, establishing hydrogen-bonding contacts with Glu376 in HSL1 and with the Asp51 in SERK1 (Supplementary Fig. 6). To assess the contribution of the C-terminal region of IDA to HSL1 binding and co-receptor sensing, we engineered a Lys66/Arg67/His68→Ala mutant and we tested its efficiency in binding assays. The triple IDA mutant showed ~15 fold reduced binding affinity to HSL1 and a ~30-fold K_*d*_ reduction in the presence of the co-receptor compared to the wild-type peptide (Fig. 2f and Fig. 1a, b and Supplementary Fig. 8). These results agree with the role of the conserved IDA C-terminus in the control of floral shedding. The triple mutant Lys66/Arg67/His68→Ala introduced in the *ida* mutant under IDA native’s promoter, was not able to complement the delay in floral organ abscission (Fig. 2g and Supplementary Fig. 6). Taken together, HSL1 can discriminate between IDA/IDLs and the CLE9 peptide by ‘reading’ structural differences in the N-terminal and core peptide regions.

LRR-RK BAM1 has been previously implicated in the perception and signaling of CLE peptides^27^, sensing CLE9 with high-specificity in the nM range^17,21,26^. To gain mechanistic insights into CLE9 recognition by BAM1, we expressed, purified and crystallized a BAM1–CLE9 complex, but could not obtain diffracting crystals. We thus built a BAM1 homology model using the CLE-receptor PHLOEM INTERCALATED WITH XYLEM (PXY) as a template (Supplementary Fig. 9, see Methods). We docked the CLE9 peptide into the BAM1 model, using high-resolution peptide modeling^38^. The docking was performed using three starting peptide orientations based on CLE9 in HSL1, IDA in HSL1 and CLE41 in PXY. Interestingly, all of them converged into a similar minimal-energy elongated peptide conformation that fully accommodated into the BAM1-model binding groove, resembling CLE41 recognition by PXY^25^ (Supplementary Fig. 9). In our BAM1-CLE9 model, the N-terminal peptide region reveals as the main recognition and docking point to the receptor with a combination of hydrophobic and electrostatic interactions matching the architecture of the receptor pocket (Supplementary Fig. 9). Different from HAE-type receptors, BAM1 has a distinct array of amino acids in its core region, with a highly conserved bulky residue (Phe269^BAM1^, Phe279^PXY^) disrupting the previously described hydroxyproline and Ser5 coordination sites in HSL1 (Fig. 2d, Supplementary Fig. 7 and Supplementary Fig. 9). Interestingly, unlike IDA/IDLs in HSL1, the central hydroxyproline in CLE9^17^ does not seem to be coordinated in the BAM1 receptor model, suggesting that is not a determinant peptide feature for receptor recognition (Supplementary Fig. 9).

**Fig. 3:**
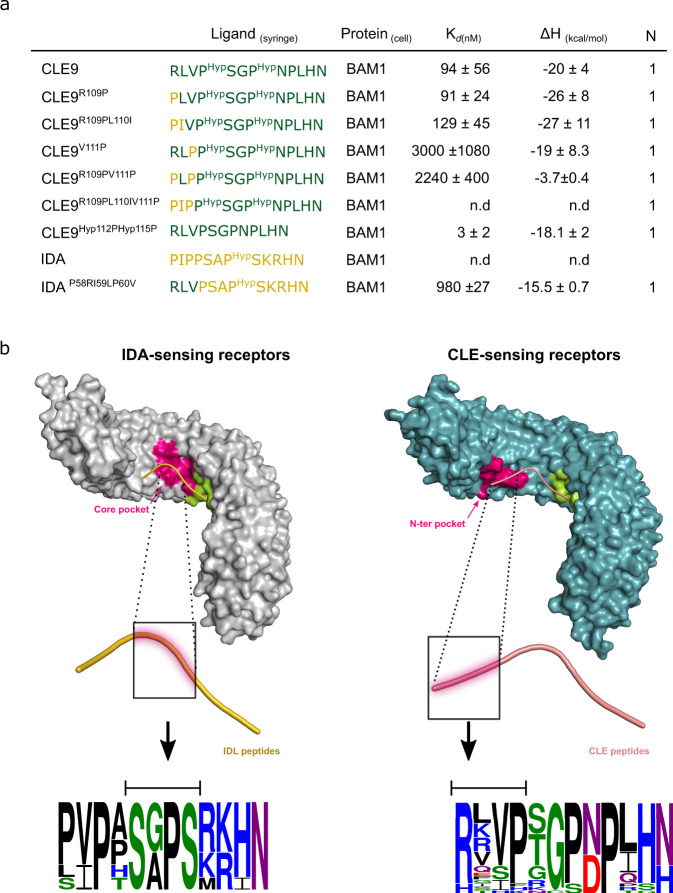
The CLE9 peptide uses its N-terminal region to specifically anchor to its cognate receptor BAM1. N-terminal engineered CLE9 peptides lose their capacity to bind the BAM1 LRR ectodomain **a** ITC table summaries of engineered CLE9 and IDA variants versus BAM1. K_*d*_, dissociation constant; N, stoichiometry (*n* = 1 for a 1:1 interaction). The values indicated in the table are the mean ± SD of at least two independent experiments. **b** Representation of the specific recognition features of IDA/IDLs and CLE-like peptides by their cognate receptors. IDA/IDL peptides and IDA-like receptors are depicted in gold (cartoon) and gray (surface), respectively. CLE-like peptides and CLE-like receptors are shown in pink (cartoon) and dark cyan (surface), respectively. The receptor region highlighted in magenta depicts unique structural features for peptide recognition in each type of receptors; and illustrates the receptor region where the peptide is highly coordinated to form a high-affinity binding pair. We termed these regions “core pocket” and “N-ter pocket” for the IDA-like and CLE-like receptors, respectively. The surface depicted in light green corresponds to a common receptor architecture, with a similar peptide-binding recognition in both types of receptors. A magnification of the peptides is shown to highlight the amino acid position at the specific receptor pocket. Peptide sequence logos for each peptide family were generated through the WebLogo 3 server (http://weblogo.threeplusone.com/^70^) using the sequence information of the IDA/IDL peptides (IDA and IDL1-6) and the mature sequence of the 27 *A. thaliana* CLE peptides^71^.The size of the amino acid letters correlates with the residue frequency, and the color code depicts the following amino acid properties: green for polar, purple for neutral, blue for basic, red for acidic and black for hydrophobic residues.

To validate the structural analysis on our BAM1-CLE9 complex docking model, we engineered CLE9 and IDA variants substituting the different N-terminal residues by the corresponding ones in IDA or CLE9, respectively, and we tested their binding capacities to the BAM1 extracellular domain. Substitutions of the first and second residues in CLE9 did not significantly impact the binding. However, replacement of the third Val by a Pro (CLE9^V111P^) dramatically reduced the binding affinity compared to the wild-type CLE9 peptide (100 nM vs 3000 nM) (Fig. 3a and Supplementary Fig. 10). Complete substitution of the N-terminal region in CLE9 (CLE9^R109PL110IV111P^) led to non-detectable binding. On the other hand, we could restore binding to BAM1 replacing the N-terminal of IDA by the one of CLE9 (IDA^P58RI59LP60V^), confirming the relevance of this region for specific peptide-receptor recognition between CLE9 and BAM1 (Fig. 3a and Supplementary Fig. 10).

We next analyzed the contribution of the CLE9 central hydroxyproline to BAM1 interaction. Replacing Hyp in positions 4 and 7 by Pro in CLE9 (CLE9^Hyp112PHyp115P^) did not impair binding to BAM1 (~5 nM and 100 nM) (Fig. 3a and Supplementary Fig. 10). Similar to HSL1 vs CLE9, we did not detect interaction between the receptor BAM1 and IDA, suggesting that both receptors discriminate between their bona-fide peptides and other dodecamer peptide families (Fig. 3a and Supplementary Fig. 10). The LRR-RKs HSL1 and BAM1 present structurally divergent ligand binding sites that allow for the selection of compatible signaling peptides. High-affinity recognition between HSL1 and IDA peptides happens through their core regions, whereas BAM1 uses its N-terminal binding site to precisely recognize CLE peptides (Fig. 3b).

**Fig. 4:**
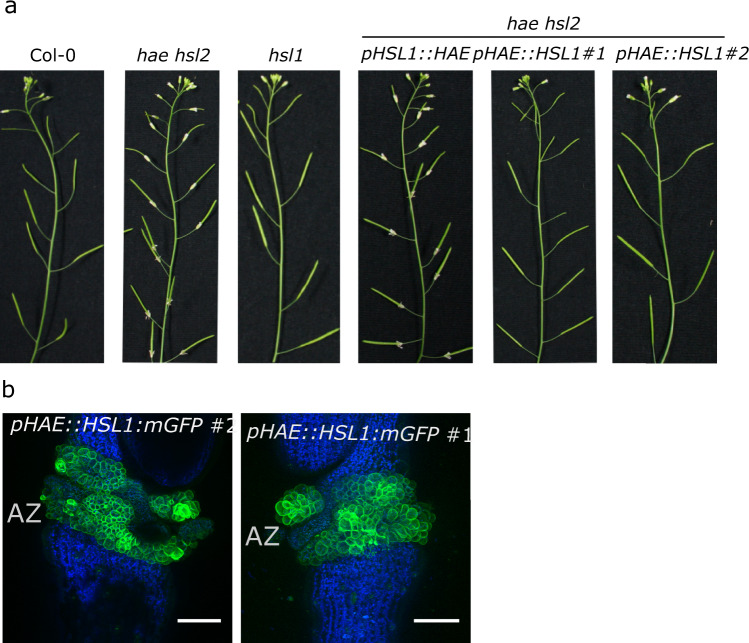
The LRR-RK HSL1 can mimic HAESA, acting as a positive regulator of floral abscission. **a** HSL1, which is not typically expressed in abscission zone (AZ), can perceive IDA in vivo and replace the receptor HAESA in floral shedding. Abscission complementation assays of *hae hsl2* double mutant expressing the receptor HSL1 under the HAESA promoter. As control, the HAESA receptor was expressed under the HSL1 promoter. A minimum of two independent lines were analyzed for floral abscission phenotype. Wilt type Col-0, and *hae hsl2* and *hsl1* mutants were also phenotyped alongside. **b** Localization of HSL1 in AZ under the expression of the HAESA promoter. Confocal images of AZ of *pHAESA::HSL1::mGFP* lines. Scale bar = 100 μm.

The above results prompted us to investigate whether HSL1 would sense IDA in vivo. To test this, we drove the expression of HSL1 in the abscission zone (AZ) using the HAESA promoter and we analyzed the recovery of floral shedding in the double mutant *hae hsl2* (Fig. 4a). Expressing HAESA under the HSL1 promoter failed to complement de *hae hsl2* mutant, suggesting that HSL1 is not typically expressed in AZ (Fig. 4a). However, the expression of HSL1 under the HAESA promoter fully restored the floral shedding phenotype, indicating that HSL1 can recognize IDA in physiological conditions and mimic the receptor HAESA, triggering the floral abscission signaling pathway (Fig, 4a-b).

**Fig. 5:**
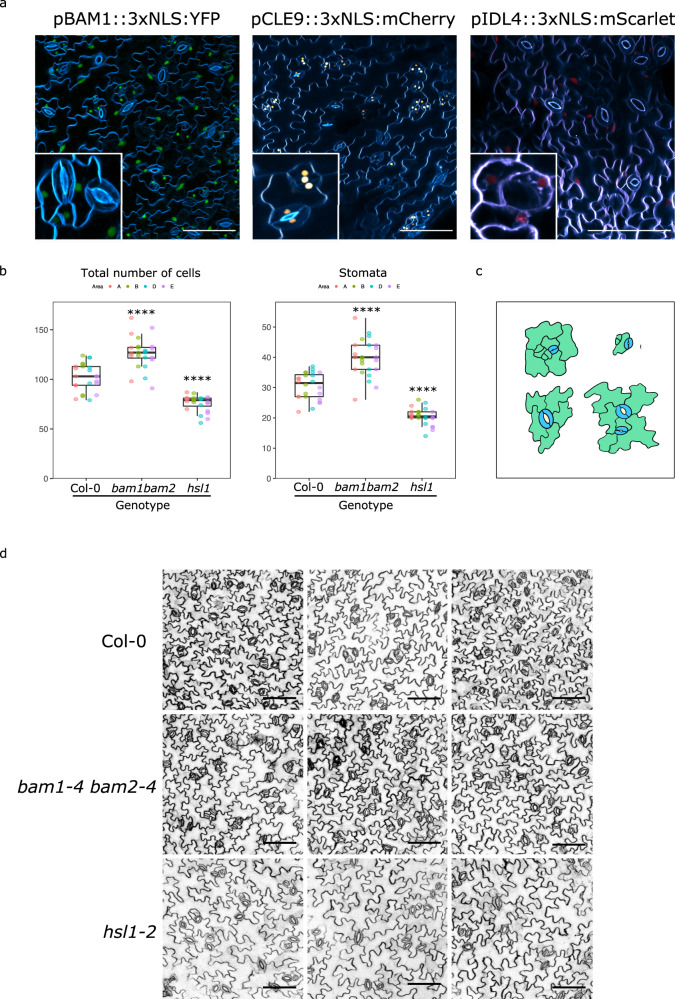
The receptors HSL1 and BAM1-BAM2 impact epidermal cell patterning via signaling of two distinct peptide families. **a** BAM1, the peptide CLE9 and IDL4 express in the same or neighboring cell types in epidermal tissue. Expression pattern of *pIDL4::3xNLS::mScarlet*, *pCLE9::3xNLS::mCherry* and *pBAM1::3xNLS::YFP* in the abaxial side of 5-day-old cotyledons. Scale bar = 100 μm **b** BAM1/2 and HSL1 regulate epidermal cell development. Effects of the double mutant *bam1-4 bam2-4 and hsl1-2* on the number of stomata and total number of cells in cotyledons of 10-day-old seedlings. Box plots representing the different total number of cells and stomata of Col-0 vs *bam1-4 bam2-4* and *hsl1-2*. * *p* < 0.05, ** *p* < 0.01, *** *p* < 0.001 and **** *p* < 0.0001 by two-sided mixed effect model with a Poisson link function and area as a random factor. The different areas counted per cotyledon are depicted in different colors. n = 2457, 3028, 1822 cells were counted over 6 independent cotyledons for Col-0, *bam1-4 bam2-4* and *hsl1-2*, respectively. The centerline of the box plots represents the median value (50th percentile), while the box contains the 25th to 75th percentiles of the dataset. The black whiskers mark the 5th and 95th percentiles, and values beyond these upper and lower bounds are considered outliers. **c** Schematic representation of epidermal cells counted in cotyledons in b. Young and well-defined stomata are depicted in blue. Non-guard cells (NGCs) are depicted in green, including pavement cells and stomata lineage ground cells (SLGCs). **d** Representative confocal images of *hs1-2, bam1-4 bam2-4,* and wild-type Col-0 cotyledons analyzed in the experiment shown in (**b**). Scale bar = 100 μm.

CLE9 and HSL1 have been reported to be expressed in leaf epidermis and to negatively regulate the proliferation of stomatal lineage cells^21,22^. Thus, we examined if BAM1 receptor would also be expressed in this tissue. *pBAM1::3xNLS::YFP* reporter lines confirmed that BAM1 is also expressed in the leaf epidermal tissue along with CLE9 (Fig. 5a). Expression of IDL4 has been reported in the guard cells (GC) of young seedlings^39^. Generation of *pIDL4::3xNLS:mScarlet* confirmed its expression in GCs, and revealed that IDL4 is also expressed in pavement and meristemoid mother cells in leaf epidermis (Fig. 5a). Next, we investigated the effect of loss-of-function *hsl1* and the double mutant *bam1 bam2* in epidermal cell division and stomata development in cotyledons. We analyzed the double mutant *bam1-4 bam2-4*^26^ in Col-0 background that displays a characteristic short root phenotype observed in other *bam1 bam2* combinations^28^ (Supplementary Fig. 11). Interestingly, the double mutant *bam1 bam2* consistently showed an increased number of cell divisions, resulting in a higher number of total cells, including stomata and non-guard cells when compared to wild-type cotyledons (Fig. 5b–d and Supplementary Fig. 11). These observations, together with our structural and biochemical analysis, suggest that BAM1/2 regulates epidermal cell division homeostasis via the sensing of CLE peptides expressed in leaves. On the other hand, *hsl1* mutant showed a reduced number of total amount of cells, mainly in stomata number (Fig. 5b–d and Supplementary Fig. 11), with enlarged pavement cells potentially compensating for the shortage in leaf cell number^40^ (Fig. 5d). These results indicate that epidermal cell development is guided by several layers of regulation mediated by different LRR-RKs and peptide hormones.

## Discussion

The organization of receptors and their cognate peptide ligands in large gene families, renders identification of bona-fide receptor–ligand pairs in plant development challenging.

Our work reveals that HSL1 is a receptor for IDA/IDL signaling peptides. By comparing the binding mode between IDA/IDLs and the sequence-related but functionally distinct CLE9 peptide, we uncover how their cognate plant LRR-RKs discriminate between the different signaling peptide families (Fig. 3b)^41^. Our structural, quantitative biochemistry, and genetic data reveal that IDA/IDL peptides use their conserved N-terminus and core regions (S-[G/A]-Hyp-S motif) to fully occupy the ligand groove of HSL1 and sequence-related HAESA receptors (Fig. 1e, Fig. 2a,d and Fig. 3b, and Supplementary Fig. 6). In contrast, structural analysis of the HSL1-CLE9-SERK1 complex reveals that CLE9 is only partially recognized by HSL1, rationalizing the reduced binding affinities when compared to IDA/IDLs (20-fold reduction) (Fig. 1a, b and Fig. 2b, c, e). Our ligand binding assays are in agreement with the previously reported binding affinities for HSL1 vs. CLE9 (38 μM), and for the association with the co-receptor SERK1 (1.5 μM)^21^.

Structural superimposition of the CLE-receptor PXY^25^ and HSL1 highlights that HAE-type and CLE-sensing receptors have evolved different peptide-binding surfaces in their LRR domain: HSL1 features a set of bulky amino acids in the N-terminal part of the binding surface, incompatible with specific CLE peptide perception. In contrast, CLE-sensing receptors contain a conserved core pocket composed of bulky residues that prevent the formation of the extended hydrogen-bonding network that we show is critical for IDA/IDL recognition (Fig. 2d, Supplementary Fig. 7, Supplementary Fig. 9).

Consistent with our previous work on HAESA^4^, the HSL1-IDA/IDL structures and genetic complementation assays show that the central hydroxyproline in IDA/IDLs is buried in the HSL1 peptide-binding groove and essential for receptor-peptide recognition and bioactivity (Fig. 2 a–d, f, g). Together, our data support that HSL1 shares a peptide recognition mechanism with other HAE-type receptors. However, HSL1 displays an extended hydrogen-bonding network in its core ligand interaction surface (Fig. 2d and Supplementary Fig. 12), accounting for the higher binding affinity to IDA/IDL peptides when compared to HAESA (Fig. 1a–d and Supplementary Fig. 2–4 and 12)^4^.

In contrast, CLE9 requires its first three N-terminal residues to be specifically recognized by its cognate receptor BAM1 (Fig. 3a, b). This distinct perception mechanism is shared among other CLE peptides^25,41^, suggesting that CLEs may use their sequence-diverse N-terminus to target specific CLE receptors (Fig. 3a, b)^41^.

Unlike IDA/IDLs, CLE peptides have been reported to carry hydroxyprolines that can be further glycosylated. This additional post-translational modification can strengthen receptor-ligand interaction^8^. In the case of CLE9, different combinations of arabinosylated peptide species have been detected in planta with no obvious contribution to receptor binding^17^. Our quantitative binding assays indicate that Hyp modifications in CLE9 are not required to anchor CLE9 to its receptor BAM1 (Fig. 3a). It is of note, that our structural and biochemical analysis support previous phylogenetic studies that classify IDA/IDL and CLV3/CLE peptides in two distinct gene families^15,42^.

This study, together with previous reports^21,39^, suggests a function for the LRR-RKs BAM1 and HSL1, and their cognate peptides ligands CLE9 and IDL4, respectively, in leaf epidermal development (Fig. 5a-d). CLE9 has been previously reported to act as a negative regulator of stomatal lineage proliferation through destabilization of the transcription factor SPEECHLESS^21^; and to signal via its cognate receptor BAM1 to control xylem file number in roots^21^. We find that leaf epidermal cell number is increased in *bam1 bam2* mutants (Fig. 5b-d), suggesting an additional role for BAM1/2 in epidermal cell division homeostasis. Qian et al. did not observe an increased number of epidermal cells in the *bam1 bam2* mutant allele used in their study. Since their allele (*bam1-3 bam2-101*) did not show the characteristic *bam1bam2* short root phenotype (Supplementary Fig. 11)^28^, we speculate that it may represent a weak allele, potentially explaining the masking of the cell division phenotype^21^.

Our work demonstrates that *hsl1* has a reduced number of stomata in the leaf epidermis (Fig. 5b,d). This suggests that HSL1 has a positive regulatory role in the control of epidermal cell patterning via the perception of IDA/IDL peptides, with IDL4 being present in the tissue (Fig. 5a, b-d)^39^. Thus, HSL1 and BAM1/2 may have antagonistic roles in leaf epidermal cell homeostasis, by specifically interacting with two distinct classes of signaling peptides.

An alternative scenario would be that HSL1 has evolved to sense IDA/IDLs and CLE9 with different binding affinities. For this scenario to be of in vivo relevance, higher concentrations of CLE9, in leaf epidermal tissue, would be required to efficiently compete with IDLs to bind HSL1. However, binding of both peptide families would result in the HSL1–SERK1 complex formation, triggering the same cytosolic signaling outputs^36,43^.

How these different receptor-ligand-co-receptor signaling complexes integrate with the well-characterized stomatal lineage and cell differentiation pathway^44,45^, will provide future mechanistic understanding of how different signaling inputs shape plant development.

## Methods

### Protein expression and purification

All genes used for protein expression in *Trichoplusia ni* Tnao38 cells^46^ were codon optimized and cloned into a modified pFastBac vector (Geneva Biotech) providing an azurocidin^47^ or Drosophila BIP signal peptide^48^, a TEV (tobacco etch virus protease) cleavage site and a Strep-9xHis tandem affinity tag at their carboxy terminus. The genes used are coding for the Arabidopsis ectodomains of HSL1 (residues 17-618, AT1G28440), HAE (residues 20-620, AT4G28490) SERK1 (residues 24–213, AT1G71830), and BAM1 (residues 20–637, AT5G65700). To perform Grating-coupled interferometry (GCI) experiments, HSL1 was fused to a non-cleavable Avi-tag^49,50^. Baculovirus generation was carried out in DH10 cells and virus amplification was done in *Spodoptera frugiperda* Sf9 cells. For protein expression, *Trichoplusia ni* Tnao38 cells were infected with HAE, HSL1, BAM1 and SERK1 virus with a multiplicity of infection (MOI) of 3. The cells were grown 1 day at 28 °C and two days at 22 °C at 110 rpm. After every expression round, secretion of our proteins was detected using an anti-His-HRP conjugated antibody from Roche (Ref:11965085001) in a 1:2000 dilution. The secreted proteins were purified separately by sequential Ni^2+^ (HisTrap excel, GE Healthcare, equilibrated in 25 mM KPi pH7.8 and 500 mM NaCl) and Strep (Strep-Tactin Superflow high-capacity, (IBA, Germany) equilibrated in 25 mM Tris pH 8.0, 250 mM NaCl, 1 mM EDTA) affinity chromatography. Next, affinity tags were removed by incubating the purified proteins with recombinant His-tagged TEV protease, in a 1:50 ratio. The cleaved tag and the protease were separated from the ectodomains by a second Ni^2+^ affinity step. Proteins were further purified by size-exclusion chromatography on a Superdex 200 Increase 10/300 GL column (GE Healthcare) equilibrated in 20 mM citric acid pH 5.0, 150 mM NaCl. Peak fractions containing the single proteins or complexes were concentrated using Amicon Ultra concentrators (Millipore, MWCO 10,000 for SERK1 and 30,000 for HAE, HSL1, and BAM1) and used for crystallization or biochemical experiments. Proteins were analyzed for purity and structural integrity by SDS-PAGE.

### Crystallization and data collection

Crystals of the isolated HSL1 ectodomain developed at 18 °C in hanging drops (vapor diffusion) composed of 1.0 μL of protein solution (12 mg/mL) and 1.0 μL of the crystallization buffer (0.2 M ammonium sulfate, 0.1 M citric acid pH 4.0, 30% w/v PEG 1000), suspended above 0.6 mL of crystallization buffer. For structure solution crystals were derivatized and cryoprotected by serial transfer into crystallization buffer supplemented with 20% ethylene glycol and snap frozen in liquid nitrogen. A 1.83 Å native dataset was collected. Ternary complexes of HSL1with the peptides IDA, IDL1, IDL2, IDL3, CLE9, and the protein SERK1, were obtained by mixing HSL1, SERK1 and the peptide hormones in a 1:1:1.5 molar ratio. Crystals from the different complexes also grew at 18 °C in hanging drops (vapor diffusion) composed of 1.0 μL of protein solution (15 mg/mL) and 1.0 μL of the crystallization buffer suspended above 0.6 mL of its corresponding crystallization buffer. The different complexes crystallized in the following conditions: crystals from HSL1-IDA-SERK1 complex grew in 0.2 M K/Na tartrate and 20% w/v PEG 3350. Crystals were cryoprotected using 15% (v/v) ethylene glycol and snap frozen in liquid nitrogen. Two datasets from the same crystal were merged and used for structure determination at 2.31 Å. Crystals from the HSL1-IDL1-SERK1 complex developed in 0.2 M ammonium citrate tribasic pH 7.0 and 20% w/v Polyethylene glycol 3350 Crystals were cryoprotected using 15% (v/v) ethylene glycol and snap frozen in liquid nitrogen. Two datasets from the same crystal were merged and used for structure determination at 2.38 Å resolution. In the case of HSL1-IDL2-SERK1 crystals, they grew in 0.2 M ammonium sulfate, 0.1 M Bis-Tris and 25% w/v PEG 3350. Crystals were cryoprotected 20% (vol/vol) ethylene glycol. A native dataset to 2.20 Å resolution was collected. Crystals of the complex HSL1-IDL3-SERK1, developed in 0.2 M MgCl_2_, 0.1 M HEPES pH 7.5 and 25% PEG 3350, and crystals were cryoprotected in 15% (v/v) ethylene glycol prior to flash freezing in liquid nitrogen. A native dataset to 2.70 Å resolution was collected. The complex HSL1-CLE9-SERK1 crystallized in 0.2 M K/Na tartrate, 20% w/v PEG 3350. Crystals were cryoprotected in 25% (v/v) ethylene glycol. A dataset to 2.87 Å resolution was collected. Data from all the crystals were collected at beam-line PXIII at the Swiss Light Source (SLS), Villigen, CH.

### Structure determination and refinement

Diffraction intensities for HSL1 apo and for the different peptide complexes were integrated and scaled with DIALS using xia2^51^ and AIMLESS^52^, respectively. The specified lattice symmetry for each crystal is indicated in Supplementary Table 1. The structure of HSL1 was determined by molecular replacement using the program PHASER, implemented in the Phenix Mrage pipeline^53^. Search models were selected using the program HHPRED^54^ and tested iteratively in the Mrage pipeline. The solution was based on LRR ectodomain of HAESA (PDB: 5IXO)^4^ and gave the asymmetric unit. Molecular replacement solutions for the IDA/IDLs and CLE9 complexes were found with Phaser-MR using the crystal structure of HAESA-IDA-SERK1 (PDB ID: 5IYX) as search template. Translational non-crystallographic symmetry (tNCS) was detected in the IDL3 crystal using Xtriage^55,56^ and a molecular replacement solution was found taking tNCS into account for this dataset. The models were manually adjusted to improve the fit to likelihood weighted electron density maps using Coot^57^. Solvent molecules were added where they were supported by both chemistry and geometry. Crystallographic refinement was performed with Refmac5^58^ using NCS for all the structures. TLS refinement was applied for the IDL2 complex. The effect of tNCS on the R factors of the IDL3 complex is reflected in the refinement statistics, Supplementary Table 1. The quality and stereochemistry of the model were evaluated using Molprobity^59^ and the wwPDB Validation Server (https://validate-rcsb-2.wwpdb.org/).

### Homology modeling and peptide docking

BAM1 LRR domain (residues 21 to 640) was used to build a homology model through the Swiss-Model homology-modeling server, using as a template the crystal structure of the receptor PXY in complex with the peptide CLE41^25^ (PDB ID 5GRQ, sequence identity 42%, coverage 92%). The docking was performed using three starting peptide orientations based on CLE41 in PXY, and CLE9 and IDA in HSL1. Final peptide-receptor docking/refinement was performed using high-resolution modeling through the Rosetta FlexPepDock tool^38^, total energy score of −428.085 and a RMSD of 0.760.

### Analytical size-exclusion (SEC) chromatography

Analytical SEC experiments were performed using a Superdex 200 Increase 10/300 GL column (GE). The columns were pre-equilibrated in 20 mM citric acid pH 5, 100 mM NaCl. 200 μg of HSL1 and the different mutant variants were incubated with SERK1 in the absence or presence of indicated peptides in a 1:1:1.5 molar ratio. Similar protein amounts were used for BAM1 SEC analysis. A non-contributing N-terminal Tyr residue was added to some of the IDA/IDL peptides to allow for peptide quantification by UV absorbance. Protein mixes were injected sequentially onto the column and eluted at 0.5 mL/min. Ultraviolet absorbance (UV) at 280 nm was used to monitor the elution of the proteins. The peak fractions were analyzed by SDS-PAGE followed by Coomassie blue staining.

### Isothermal titration calorimetry (ITC)

Experiments were performed at 25 °C using a MicroCal PEAQ-ITC (Malvern Instruments) with a 200 µL standard cell and a 40 μL titration syringe. HSL1, BAM1, and SERK1 proteins were gel-filtrated into the ITC buffer (20 mM sodium citrate pH 5.0, 150 mM NaCl). Molar protein concentrations for HSL1, BAM1, and SERK1 were calculated using their molar extinction coefficient and a molecular weight of 74,896 and 27,551 Da, respectively as previously determined^4^. A typical experiment consisted of injecting 2 μL of a range between 75 and 600 μM solution of ligand into 10 to 30 μM HAE, HSL1, or BAM1 solution in the cell at 150 s intervals and 500 rpm stirring speed. The SERK1 vs HAE/HSL1-peptide experiments were performed by titrating 100 µM SERK1 in the cell, using the same injection pattern. ITC data were corrected for the heat of dilution by subtracting the mixing enthalpies for titrant solution injections into protein-free ITC buffer. Experiments were done in duplicates or triplicates, otherwise specified, and data were analyzed using the MicroCal PEAQ-ITC Analysis Software provided by the manufacturer. All ITC runs used for data analysis have an N ranging from 0.7 to 1.3. The N values were fitted to 1 in the analysis. For binding experiments, a Tyr (Y) residue was introduced in some IDA/IDL peptides to facilitate concentration measurements. We tested and confirmed that the addition of this extra residue in the N-terminal does not affect IDA/IDLs binding capacity to HAE-type receptors^4^ (Supplementary Table 2).

### Protein Biotinylation

25 μM of HSL1 were biotinylated with the biotin ligase BirA^60^ (2 μM) for 1 h at 30 °C, in a volume of 200 μl. The buffer used in the reaction was: 25 mM Tris pH 8, 150 mM NaCl, 5 mM MgCl2, 2 mM 2-Mercaptoethanol, 0.15 mM Biotin, 2 mM ATP. Biotinylated proteins were purified by size-exclusion chromatography in 20 mM citric acid pH5, 150 mM NaCl and subsequently used in GCI experiments.

### Grating-coupled interferometry binding assays

GCI experiments were performed with the Creoptix WAVE system (Creoptix AG, Switzerland) using 4PCP WAVE chips (thin quasiplanar polycarboxylate surface; Creoptix, Switzerland). Chips were first conditioned with borate buffer (100 mM sodium borate pH 9.0, 1 M NaCl; Xantec, Germany). Chips were activated with 1:1 mix of 400 mM *N*-(3-dimethylaminopropyl)-*N*′-ethylcarbodiimide hydrochloride and 100 mM N-hydroxysuccinimide (Xantec, Germany). Streptavidin (50 µg/ml; Sigma, Germany) in 10 mM sodium acetate pH 5.0 (Sigma, Germany) was immobilized on the chip surface until saturation was reached, followed by passivation of the surface with BSA (50 µg/ml; Sigma, Germany) in 10 mM sodium acetate pH5.0, and a final quenching step with 1 M ethanolamine pH 8.0 (Xantec, Germany). Then, biotinylated HSL1 (ligand) (80–100 μg/ml) was captured on the streptavidin surface until the desired density was reached. Normal kinetic analysis were performed to measure the binding of IDL peptides (analyte) to HSL1. Experiments were performed at 25 °C with a 1:2 dilution series from a maximum concentration of 5–50 μM peptide, in 20 mM citrate pH 5, 250 mM NaCl, 0.005% Tween-20 v/v. To analyze the interaction kinetics of the co-receptor SERK1, 2 µM of the protein was used as a higher dilution with running buffer containing 5 µM of the IDL peptides being tested. Blank injections were used for double referencing and a dimethylsulfoxide (DMSO) calibration curve for bulk correction. Analysis and correction of the obtained data were performed using the Creoptix WAVE control software (correction applied: X and Y offset; DMSO calibration; double referencing). One-to-one binding model was used for both experiments.

### Plant material and generation of transgenic lines

The genotypes used in this study: *hae hsl2*^39^, *bam1-4 bam2-4*^26^*, ida-2* (SALK_133209) 11, and *hsl1-2* (SAIL_563_E08) were in Columbia (Col-0) background, used as wild-type control. Homozygosity was verified by PCR using the primers in Supplementary Table 3. Seed sterilization was done by using a vapor phase with HCl to a final concentration of 2% and bleach for 2.5 hrs. Plants were plated on ½ MS (Murashige & Skoog) media with 1% plant agar and pH 5.8, vernalised at 4 °C for 4 days and grown vertically under long-day conditions (16 h light with 23 °C and 8 h dark with 21 °C). For phenotyping, plants were grown at 22 °C with 16 h light and 8 h dark. For the generation of transgenic lines, pGGZ003 final destination vector (Addgene ID: 48869) was used for GreenGate method^61^. When the removal of the BsaI restriction site was not possible the multisite Gateway technology (ThermoFisher Scientific, MA, USA) was used. Promoters were amplified from *Arabidopsis thaliana* (Col-0 ecotype) genomic DNA and the genes coding sequences were cloned from Arabidopsis cDNA: HAESA (AT4G28490), HSL1 (AT1G28440), BAM1 (AT5G65700), IDA (AT1G68765), IDL4 (AT3G18715) and CLE9 (AT1G26600). Plasmids were built using the Greengate Cloning system^61^. Structure-based mutations were introduced in the gene sequence by site-directed mutagenesis^62^. All transgenic lines were generated using the floral dip method^63^ with the *Agrobacterium tumefaciens* strain GV3101 or ASE (pSOUP^+^).

### RNA analyses

Plants were grown on ½ Murashige and Skoog (MS) plates with 1% plant agar. After 5 days, 100 mg of plant tissue was collected and frozen in liquid nitrogen. Total RNA was extracted using a RNeasy plant mini kit (Qiagen, Valencia, CA), and the reverse transcription was done using a SuperScript IV Reverse Transcriptase (ThermoFisher Scientific, MA, USA). RT-qPCR amplifications and measurements were performed using a QuantStudio 3 Real Time PCR-System by ThermoFisher Scientific. RT-qPCR amplifications were monitored using qPCR Brilliant II SYBR Mix (Agilent). The 2−ΔΔCT (or comparative CT) method^64^ was used to calculate the relative quantification of gene expression, while ACTIN2 gene was used as a housekeeping gene. All the specific primers used for detection and amplification are listed in Supplementary Table 3. The data were collected from three independent technical experiments.

### Transient protein expression in tobacco leaves

The transient expression experiments were done in three weeks old *Nicotiana benthamiana* plants. *Agrobacterium tumefaciens* cultures were grown in LB media at 28˚C overnight. The induction mixture contained 200 µL of the *Agrobacterium* culture (OD 0.1) and 200 µM of acetosyringone in 5 mL of fresh infiltration media (50 mM MES, 2 mM NaH_2_PO_4_, 0,5% (w/v) saccharose, pH 5.6, salt mixture containing: NH_4_Cl, MgSO_4_, KCl, CaCl_2_, FeSO_4_). The mix was let on a shaker for 6-8 hrs, at 28˚C until reaching OD 0.2-0.3. For co-infiltration of two constructs, infiltration media with *Agrobacterium* harboring the desired constructs were mixed in a 1:1 v/v ratio. The infiltrated plant material was imaged and collected after 2 days post infiltration. Leaves were cut and flash frozen in liquid nitrogen. HAESA wild-type and mutant ectodomains were tested for proper plasma membrane localization in transient expression driven by 35S promoter^65^.

### Protein localization in tobacco

Transient expression of constructs containing *p35S::HAE:mVenus* and mutants were imaged on a Leica SP8 microscope. The excitation and emission wavelengths used were 514 nm and 525-550 nm, respectively. For the plasma membrane marker FM4-64 (ThermoFisher Scientific) the excitation wavelength was 515 nm and the emission was 640 nm.

### Gene expression in Arabidopsis

For protein expression pattern and localization in Arabidopsis stable lines, the following settings were used to obtain specific fluorescence signals: EGFP-ex: 470 nm/em: 490–515 nm, YFP or mVenus-ex: 514 nm/em: 520–550 nm, mCherry- ex: 594 nm/ em: 600–650 nm. Images were adjusted using ImageJ^66^.

### Quantification of epidermal and stomatal cells, and statistical analysis

Plants were plated on ½ MS (Murashige & Skoog) media with 1% plant agar and pH 5.8, vernalized at 4 °C for 4 days and grown vertically under long-day conditions (16 h light with 23 °C and 8 h dark with 21 °C). Experiments were replicated in the presence and absence of 1% sucrose. All the stomata data were acquired with Leica SP8 microscope. As membrane marker for defining the cell margins, plant material was stained using FM 4-64 (ThermoFisher Scientific) in vacuum conditions as described in^67^. Data were collected from 11 days old seedlings from the abaxial side of cotyledon’s epidermal cells. In each cotyledon, 4 regions of 0.18 mm^2^ were defined (A, B, D, and E), two on the left side of main nervure and two on the opposite side. These regions were used as sub-sampling in statistical analysis. All the cells, classified as stomata and non-guard cells (NGCs), were counted with the Fiji software^66^. For each genotype, 6 independent seedlings were used and an average of 400 cells/cotyledon for Col-0, 500 cells/cotyledon for *bam1 bam2* and 300 cell/cotyledon for *hsl1* were counted in total, in the the four regions per plant. Statistical analyses of the data were performed using a mixed-effect model with a Poisson link function and region as sub-sampling random factor for multiple, independent count endpoints in R (version 4.03). Experiment reproducibility by means of a second study was assessed using a reverse Bayesian approach^67^ with prior-predictive tail probabilities to define reproducibility success. The method provides a quantitative measure for replication success known as the skeptical p-value (*p*^*skeptical*^)^68,69^. *p*^*skeptical*^ values of 9.6e-07, 6.2e−06 and 4.17e−05 were calculated for the mutant *bam1 bam2* for total amount of cells, NGC and stomata, respectively. A similar reproducibility study was done for the *hsl1* mutant with *p*^*sceptical*^ values of 6.6e-04 for total number of cells, 0.015 for NGCs and 2.1e−04 for stomata. These values for both mutants indicate that these results are reproducible.

### Reporting summary

Further information on research design is available in the Nature Research Reporting Summary linked to this article.

## Supplementary information

Supplementary Information

Peer Review File

Reporting Summary

## Data Availability

The raw data supporting the findings of this paper are available in the provided Source data file. Materials from this paper are available through the corresponding author upon request. The crystal structures analyzed in this paper are deposited in the Protein data bank with PDB IDs: apo-HSL1 (7ODK), HSL1-IDA-SERK1 (7ODV), HSL1-IDL1-SERK1 (7OGO), HSL1-IDL2-SERK1 (7OGQ), HSL1-IDL3-SERK1 (7OGZ), HSL1-CLE9-SERK1 (7OGU). Source data are provided with this paper.

## Source data

Source Data
