# Supplementary material for: HSL1 and BAM1/2 impact epidermal cell development by sensing distinct signaling peptides
Source: Nat Commun. 2022 Feb 15;13:876. doi: 10.1038/s41467-022-28558-4 (PMC8847575; doi:10.1038/s41467-022-28558-4)
Supplement: Supplementary file 1 — Supplementary Information [file 41467_2022_28558_MOESM1_ESM.pdf]

Supplementary information

HSL1 and BAM1/2 impact epidermal cell development by sensing distinct signaling peptides.

Andra-Octavia Roman[1‡], Pedro Jimenez-Sandoval[1‡], Sebastian Augustin[1], Caroline Broyart[1], Ludwig Hothorn[2,3], Julia Santiago[1*]

[1]*The Plant Signaling Mechanisms Laboratory, Department of Plant Molecular Biology, University of Lausanne, 1015 Lausanne, Switzerland.*

[2]*Institute of Biostatistics, Leibniz University, 30167 Hannover, Germany.*
[3] *Ludwig A. Hothorn is retired.*

[*]Corresponding author: julia.santiago@unil.ch (J.S)

[‡]These authors contributed equally to this work

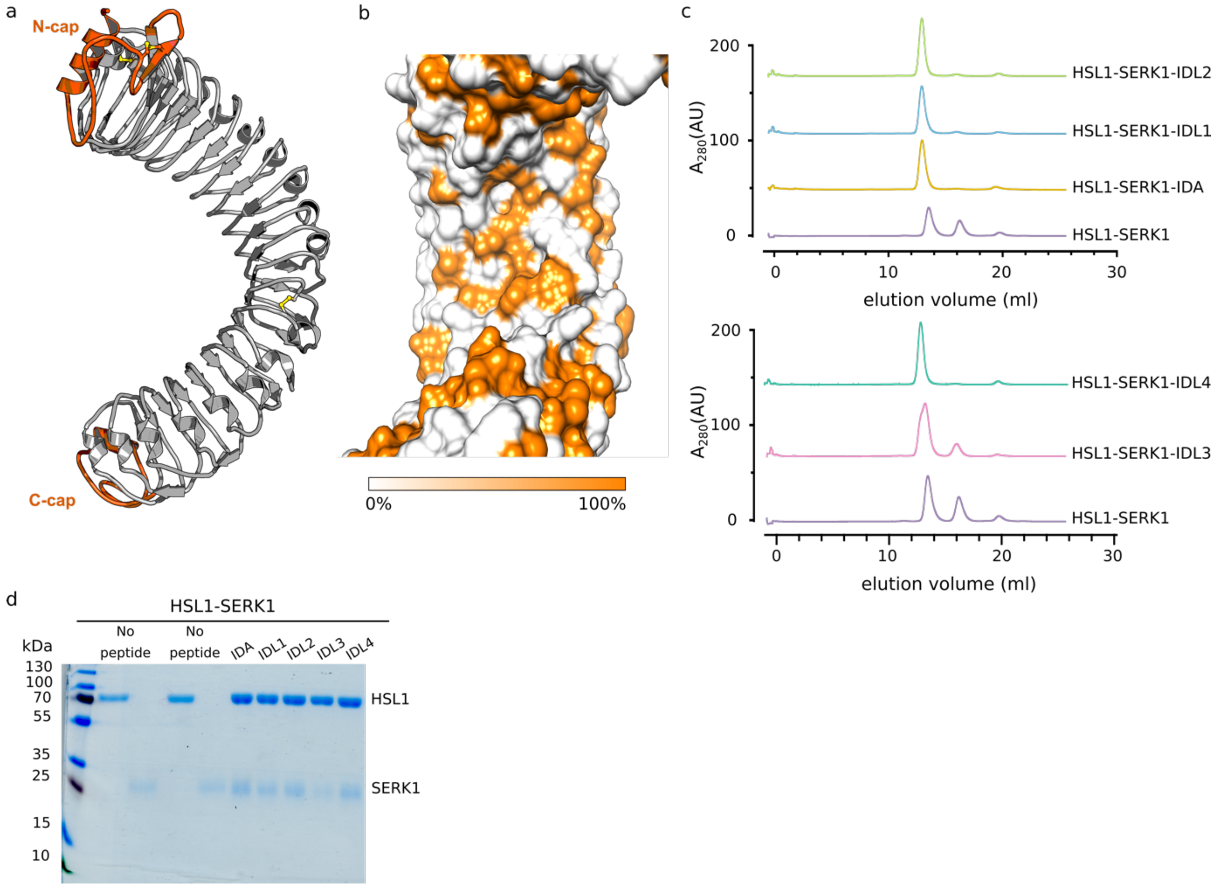

Supplementary Figure 1. HSL1 has a conserved peptide binding surface with other HAE-type receptors, and forms stable complexes with the co-receptor SERK1 in the presence of IDA/IDL peptides. a Apo structure of the LRR ectodomain of HSL1 receptor in ribbon diagram. The N- and C-terminal capping domains are shown in orange. **b** The HSL1 ligand binding surface contains key structural features to recognize IDA/IDL peptides. Binding pocket conservation analysis between HSL1 and HAE-type receptors. **c** Analytical size-exclusion chromatography experiments (SEC), in the presence of IDA/IDL peptides, reveal a stable complex formation between HSL1 and SERK1 ectodomains. **d** SDS-PAGE of the complex peaks of the SEC analysis in **c**.

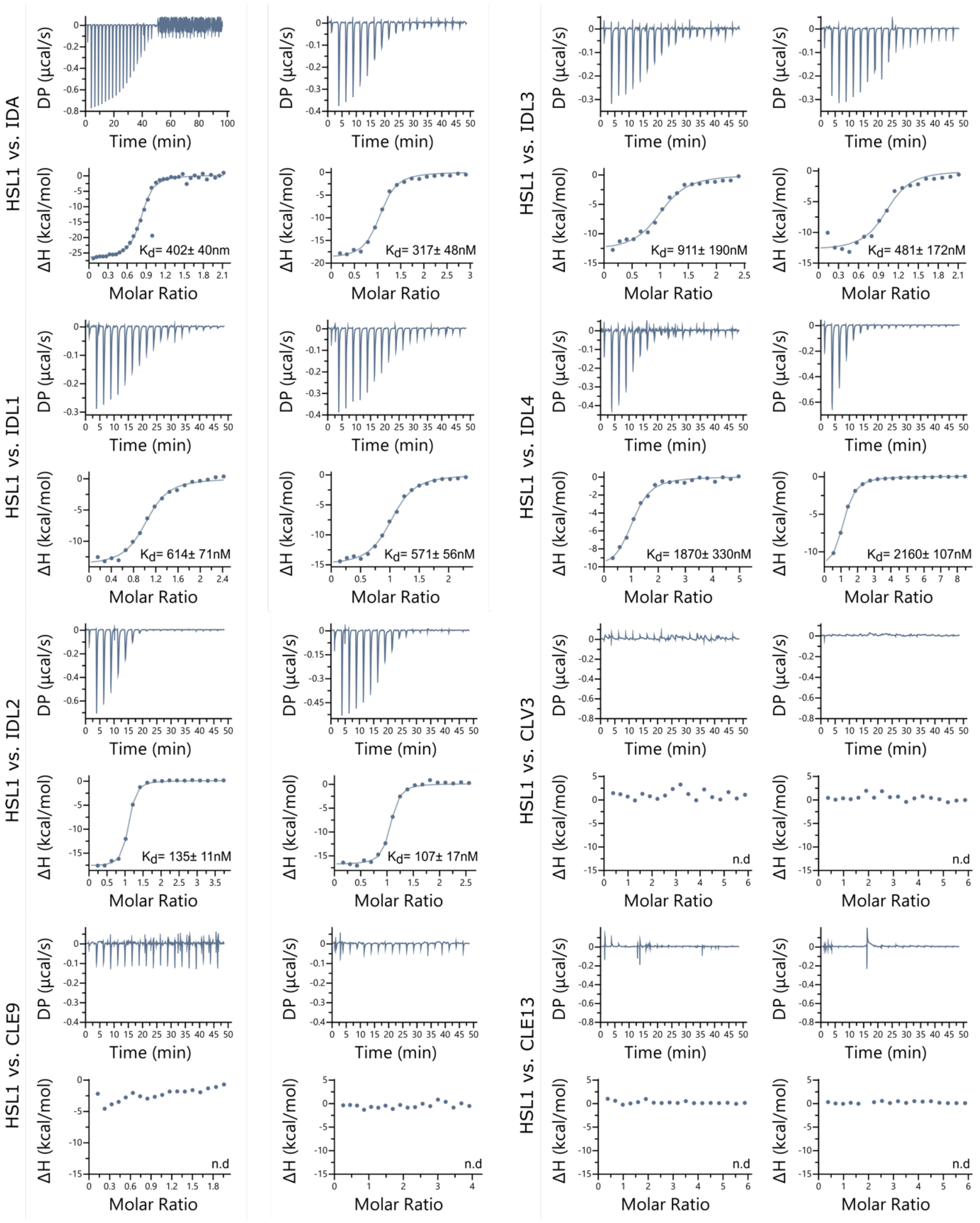

Supplementary Figure 2. Isothermal titration calorimetry assays of HSL1 vs. IDLs and CLE9. ITC thermograms of the independent experiments performed for each peptide and analyzed in Fig. 1a.

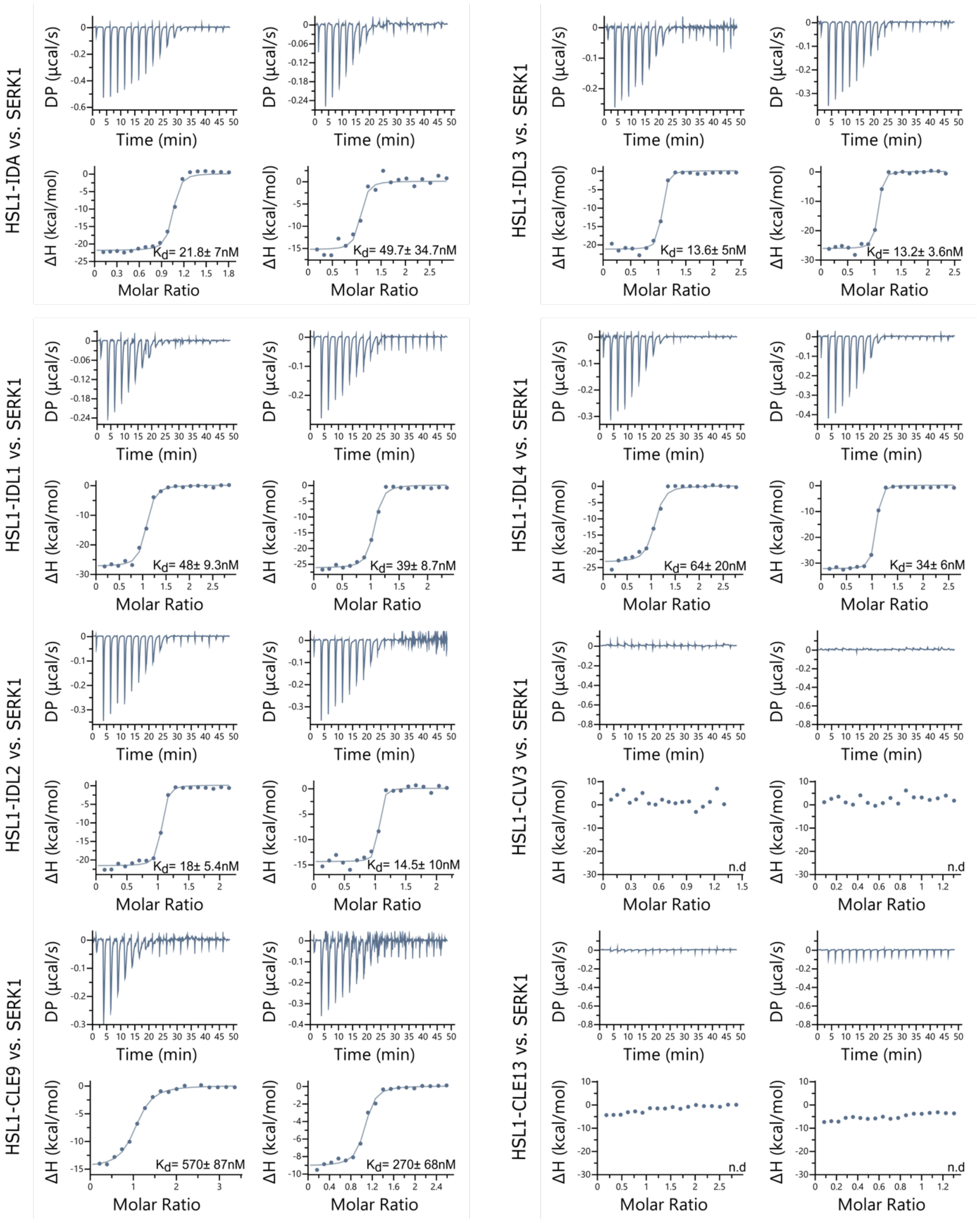

Supplementary Figure 3. Isothermal titration calorimetry assays of SERK1 binding contribution to the different HSL1-IDA/IDL and CLE9 complexes. ITC thermograms of the independent experiments performed for each peptide and analyzed in Fig. 1b.

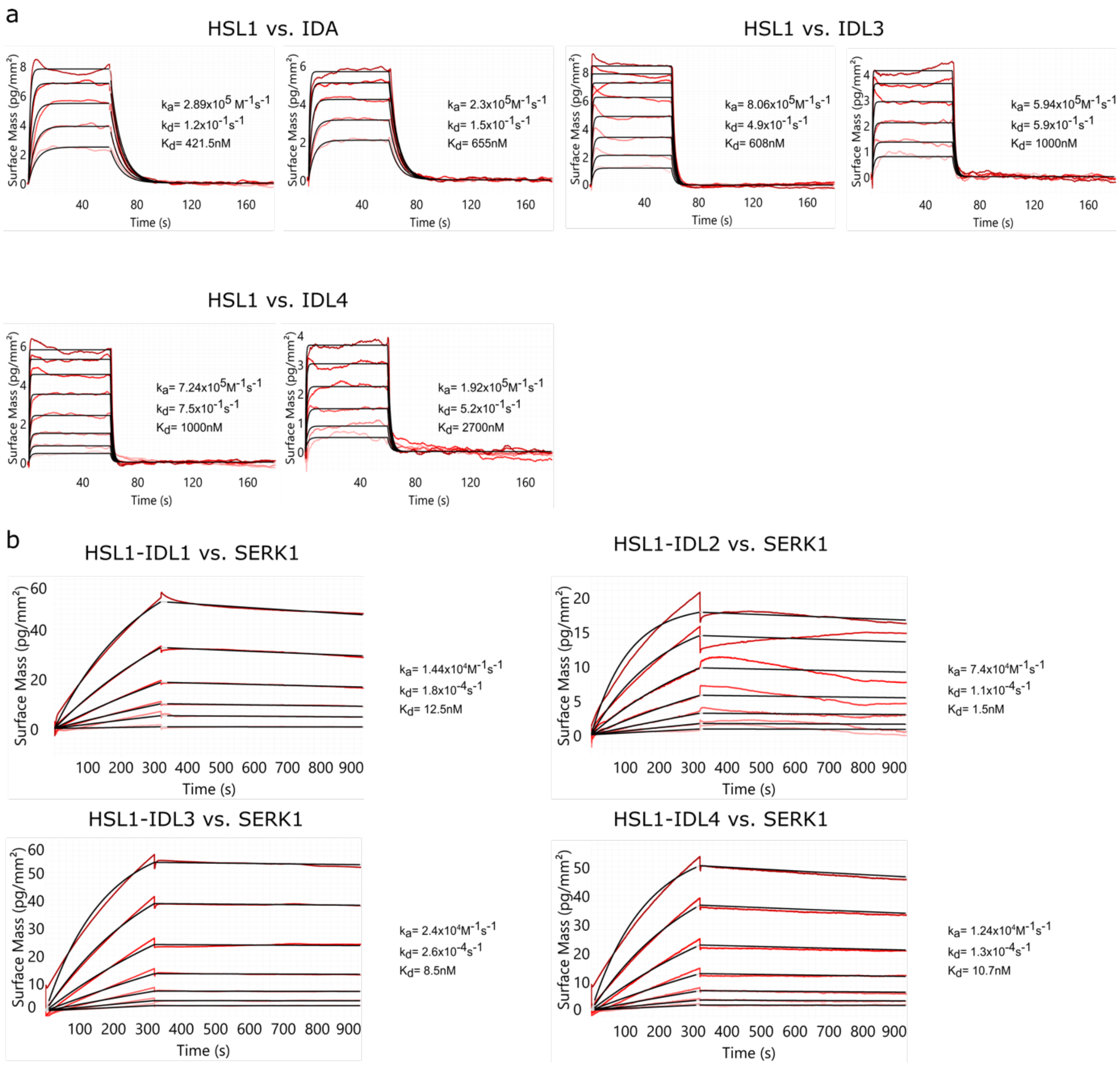

Supplementary Figure 4. Grating-couple interferometry (GCI) kinetic experiments of HSL1 vs IDA/IDL peptides and contribution of the coreceptor SERK1. a GCI sensograms of the independent experiments for different IDA/IDL peptides and kinetics of SERK1 binding to different HSL1-IDL complexes analyzed in Fig. 1d. The sensograms show the data in red and the respective curve fits in black.

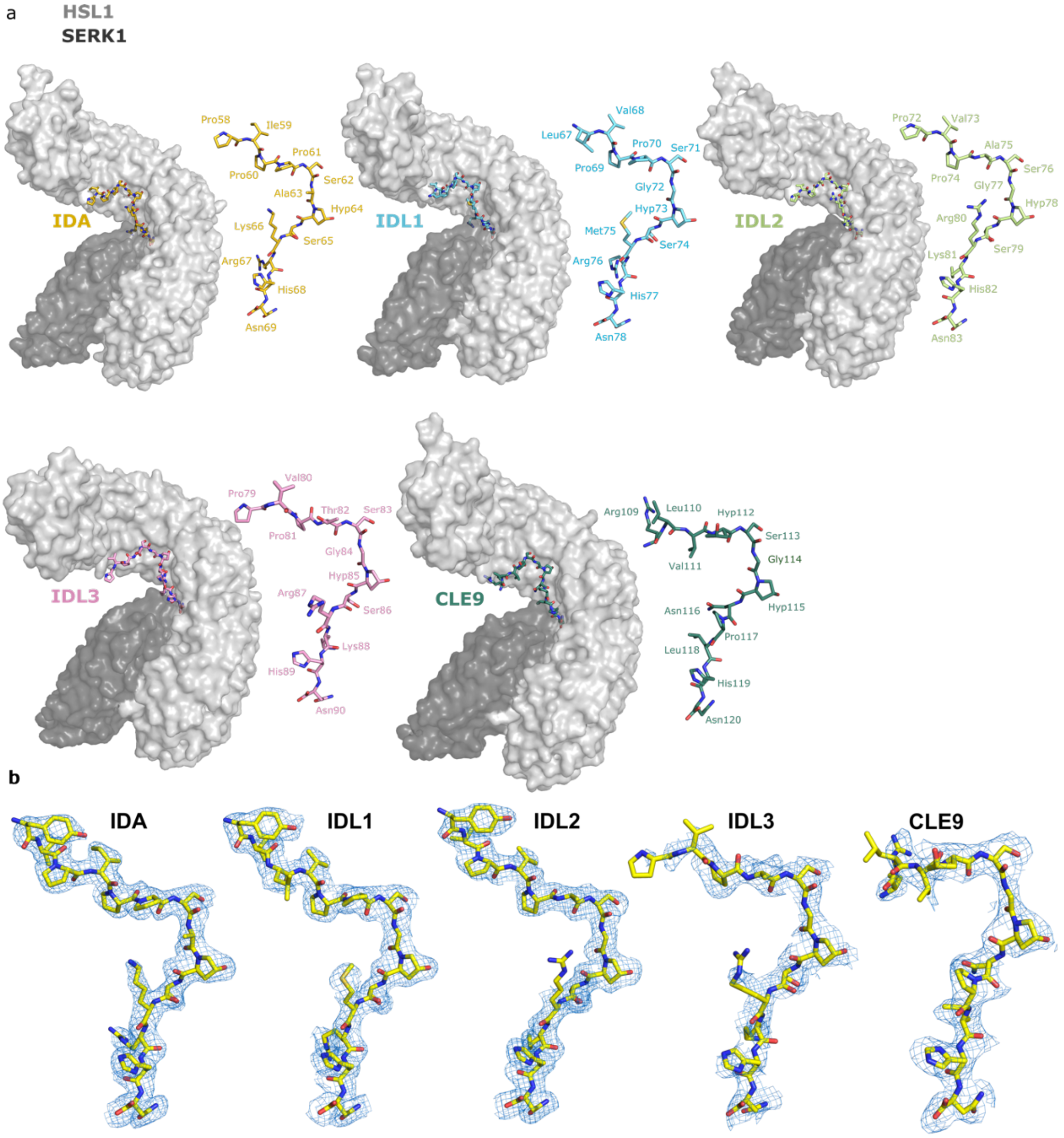

Supplementary Figure 5. Overall structures of the ternary HSL1-IDA/IDL-SERK1 and HSL1-CLE9-SERK1 complexes. a HSL1 and SERK1 are depicted in surface representation in light and dark grey, respectively. The structure of the different IDA/IDL and CLE9 peptides with the amino acid number is shown in bond representation with a different color code: IDA (yellow), IDL1 (blue), IDL2 (light green) and IDL3 (pink) and CLE9 (dark green). **b** The fully elongated conformation of IDA/IDL peptides vs CLE9 when bound to HSL1, is supported by well-defined electron density. Omit maps of the different peptides bound to HSL1 receptor. Peptides are shown in sticks (bonds representation, in yellow). The blue mesh represents the electron density map (|Fo|−|Fc| omit maps) contoured at 2.0σ.

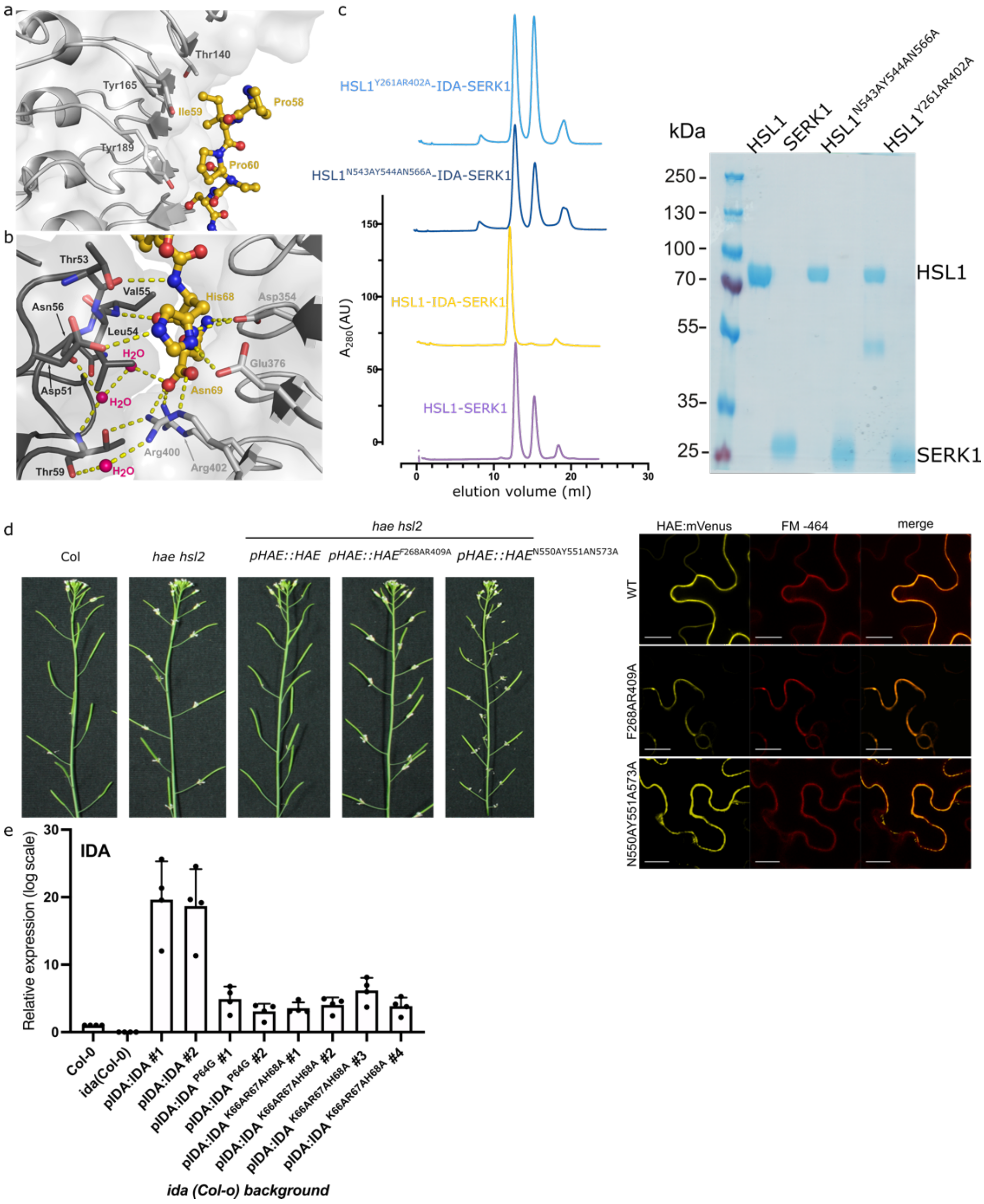

Supplementary Figure 6. Structure model validation of HSL1-IDA/IDL-SERK1 complex. a The hydrophobic N-terminal of IDA/IDL peptides accommodate into the shape-complementary HSL1 binding pocket. Zoom in view of the N-terminal of IDA bound to HSL1 ligand surface groove. The conserved third Pro (Pro60^{IDA}) orients the N-terminal of the peptide along the hydrophobic cavity composed by two tyrosines (165 and 189). **b** The co-receptor SERK1 contributes to peptide recognition using its N-terminal loop to interact with the C-terminal of IDA. The side chain of His68 in IDA bridges the interaction between the receptor and the co-receptor. Additional backbone and water mediated contact points contribute to the high affinity perception of IDA/IDLs by HSL1 and SERK1. **c** Size exclusion chromatography experiments of HSL1 mutants targeting the ligand binding surface (HSL1^{Y261R402A}) and the zipper-like interface with SERK1 (HSL1^{N543AY544AN566A}), disrupt the ternary complex formation. Right panel, SDS-PAGE of the peak fractions. Lines 1 and 2 correspond to HSL1 and SERK1 ectodomains, respectively. **d** Corresponding mutations in the HAESA binding surface, fail to form a stable signaling complex to control floral abscission. Complementation assays of *haehsl2* double mutant with HAESA wild type and HAESA mutants HAE^{F268AR409A} (ligand binding surface) and HAE^{N550AY551AN573A} (interface with SERK1). Right panel, plasma membrane localization of HAE mutants. Confocal images of *N.benthamiana* leaves transiently transformed with mVenus tagged HAE wild type and mutants. FM-464 was used as a plasma membrane marker. Scale bar = 20 µm. **e** Relative expression of IDA wild type and mutants in the independent lines analysed in *ida* complementation experiments. Transcript levels represent means ± SD of three independent experiments represented in black dots.

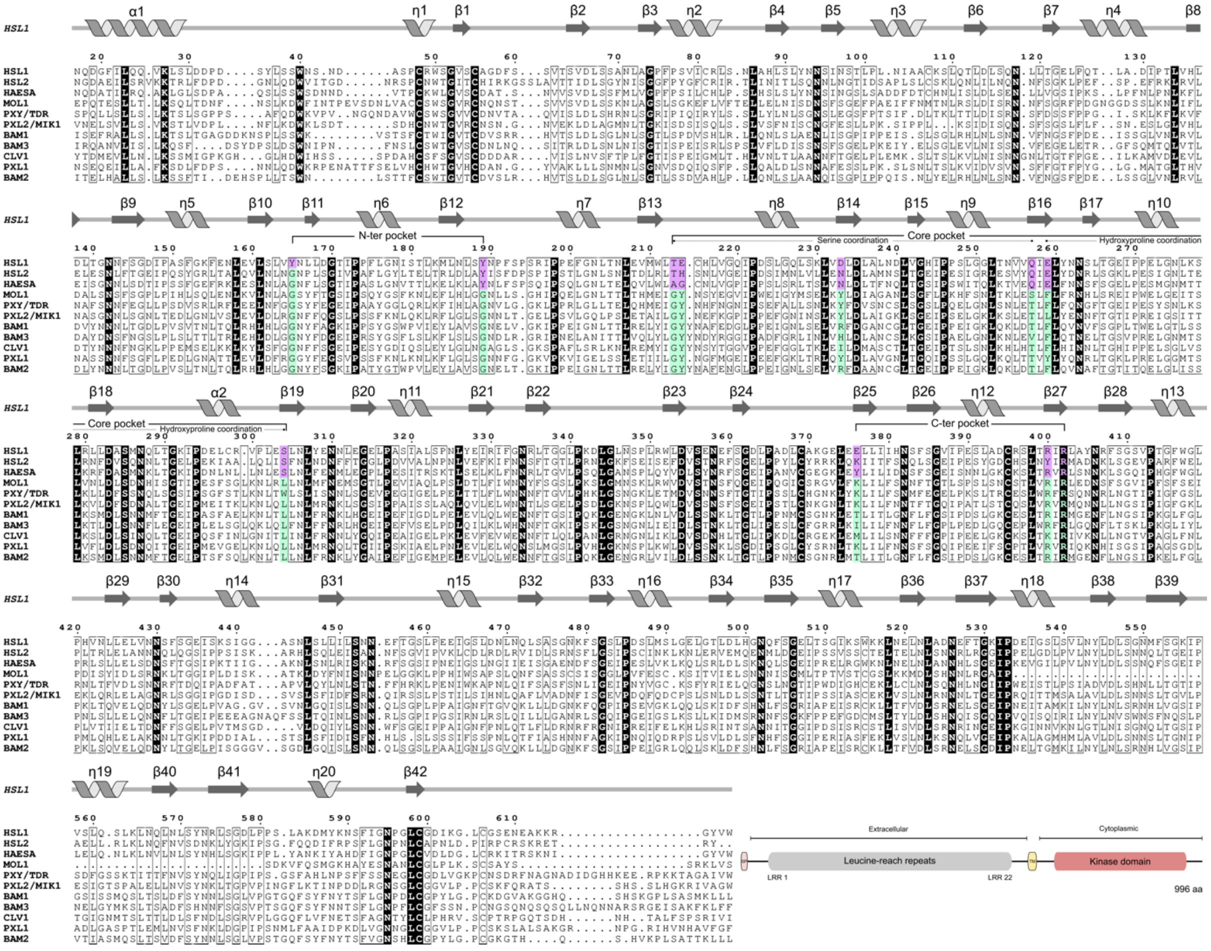

Supplementary Figure 7. Structural signatures for discriminatory recognition of IDA/IDLs and CLE peptides by HAE-like and CLE receptors are conserved among the different receptor clades. Structure-based sequence alignment of HAE-like and reported CLE receptors. The alignment includes a secondary structure assignment calculated by the program DSSP. Residues involved in the specific recognition of IDA/IDL or CLE peptides by their cognate receptors are depicted in lilac and green, respectively. A schematic of the overall receptor domain structure is represented at the bottom.

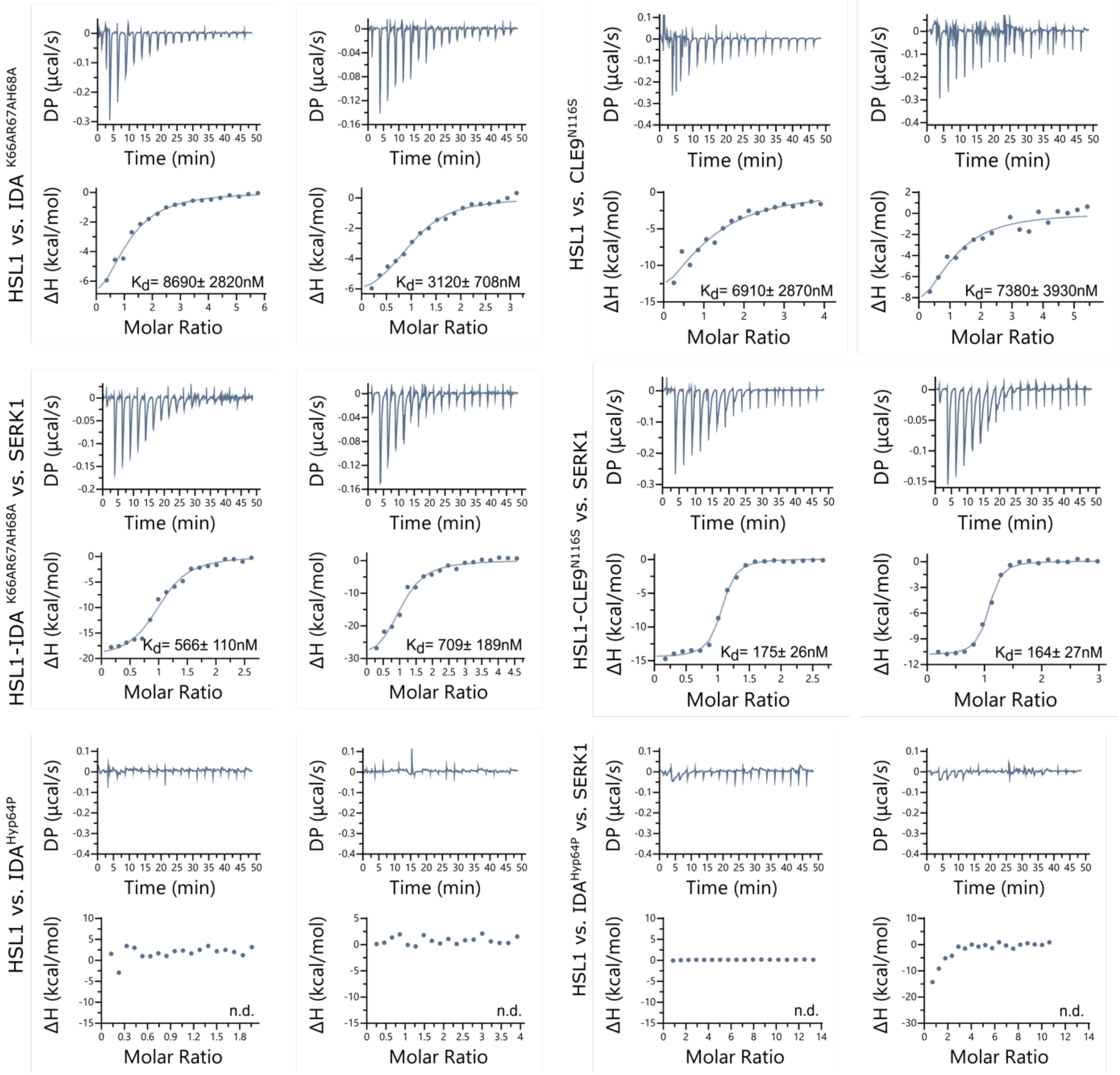

Supplementary Figure 8. Isothermal titration calorimetry thermograms of HSL1 vs IDA^{Hyp64P}, the C-terminus mutant IDA^{K66AR67AH68A} and the CLE9^{N116S} variants, and contribution of SERK1 to the complex formation. ITC thermograms of the independent experiments analyzed in Fig. 2f.

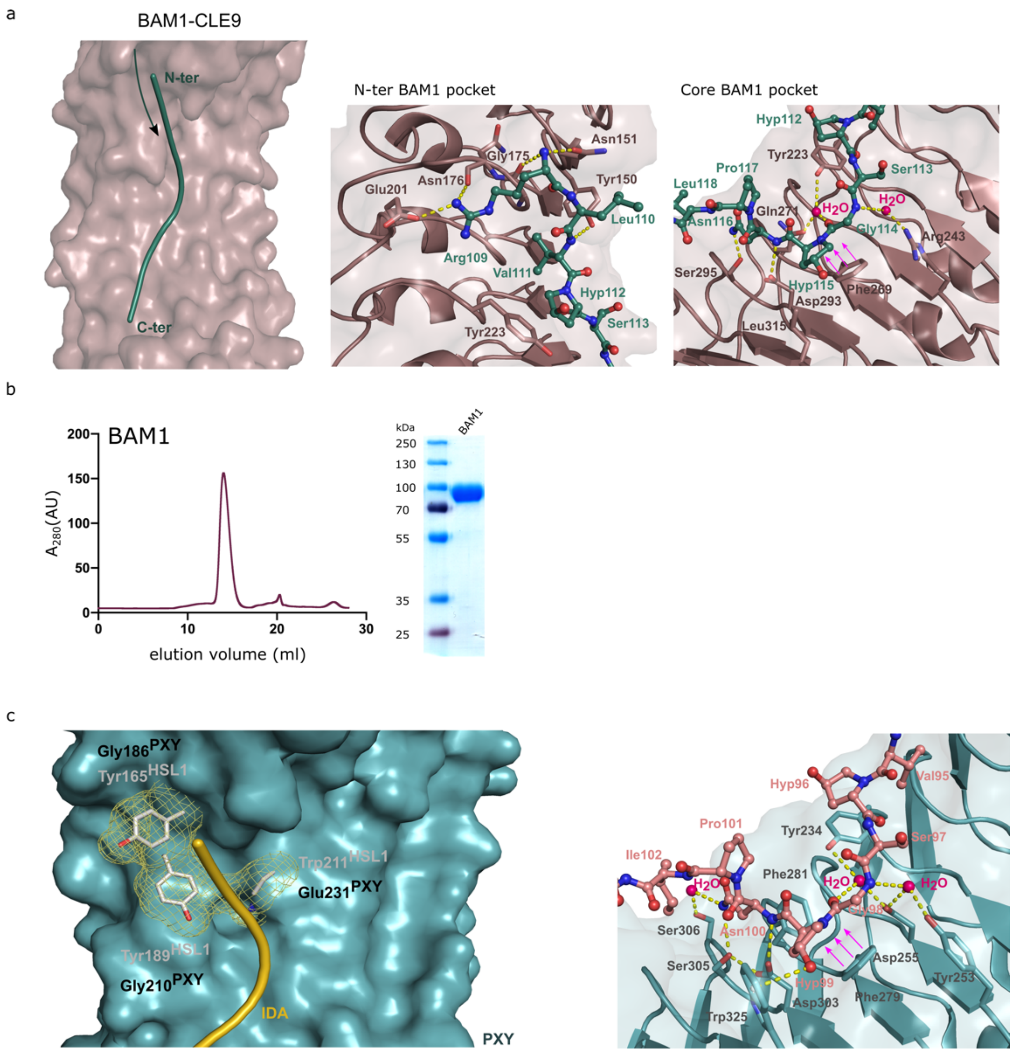

Supplementary Figure 9. BAM1 specifically accommodates the peptide CLE9 in its binding surface and shares similar structural features with other CLE receptors. a Molecular docking of BAM1-CLE9 complex. Left panel, CLE9 cartoon representation (green) along the BAM1 binding surface (magenta). The green arrow indicates the direction of BAM1 binding groove. Middle panel, the N-terminal of CLE9 anchors the peptide to the receptor. Close-up view of the N-terminal of CLE9 (bond representation in green) and BAM1 pocket (in magenta) interactions. Right panel, the core region of CLE9 does not play a major role in attaching the peptide to the receptor. Close-up view of the core region of CLE9 interactions with BAM1 binding surface. Hydrogen bonds are depicted as dotted lines in yellow and waters are shown as bright pink spheres. **b** Protein integrity and purity of the recombinantly expressed ectodomain of BAM1 LRR-RK used in the binding experiments. SEC analysis of BAM1 ectodomain. SDS-PAGE of the purified ectodomain. **c** N-terminal specific peptide recognition signatures between HSL1 and the CLE receptor PXY. Left, close-up view of the superimposed N-terminal pocket of PXY (dark cyan) and HSL1-IDA complex. HSL1 displays an array of bulky residues that accommodate the Pro-rich n-terminal part of IDA/IDL peptides. Superimposed HSL1 residues are shown in gray stick representation and surrounded by a yellow density mesh. IDA peptide is shown in gold. Right, zoom in view of the core pocket of PXY in complex with CLE41. The bulky residue Phe279 pushes the peptide to allow for the N-terminal region of the peptide to reach the receptor's N-terminal pocket. Polar contacts between the receptors (dark cyan) and the peptide (pink sticks) are depicted in yellow dash lines. Crystallographic waters are shown in hot pink spheres.

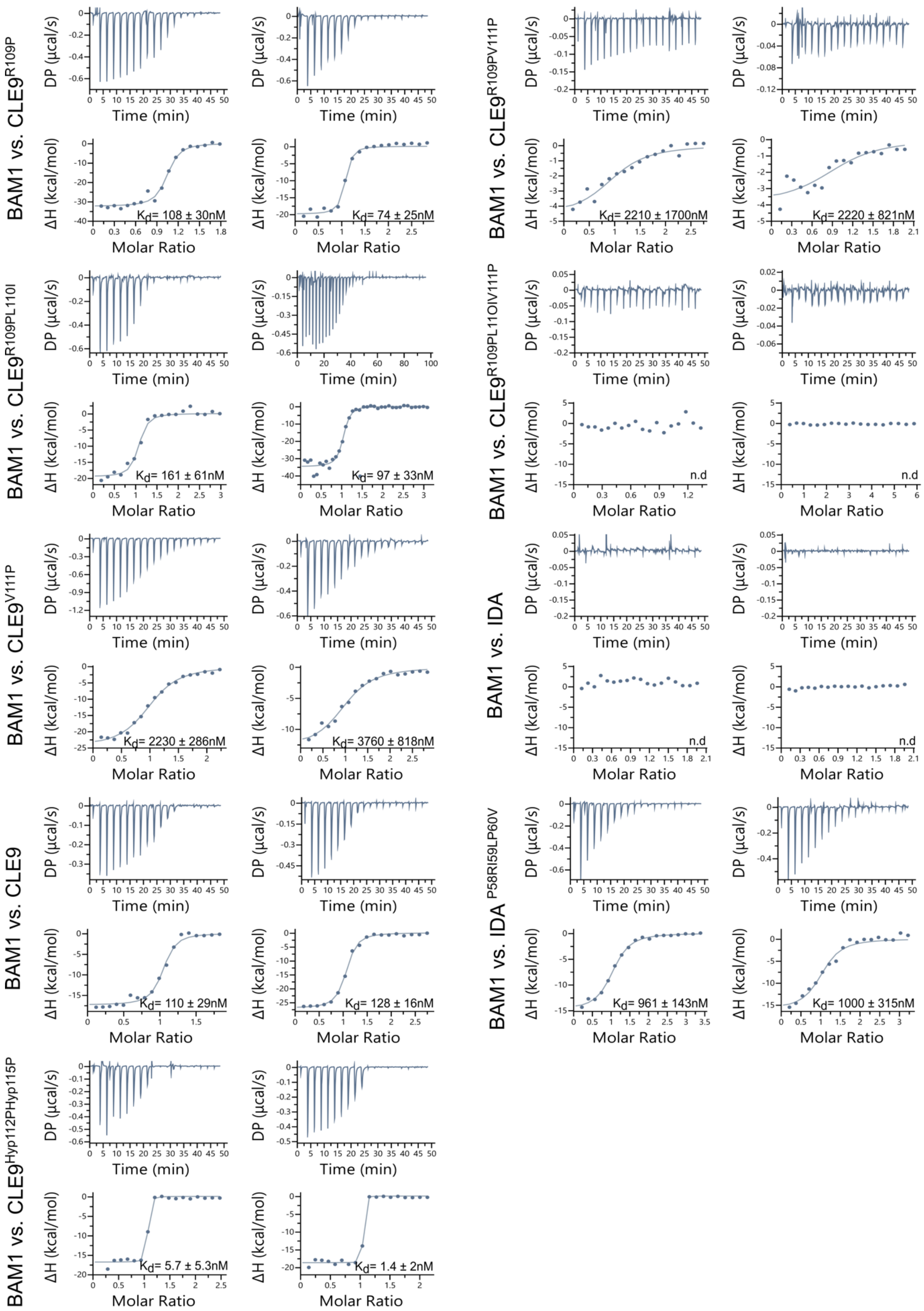

Supplementary Figure 10. Isothermal titration calorimetry thermograms of IDA, CLE9 and engineered CLE9 peptides vs BAM1. ITC thermograms of the independent experiments analysed in Fig. 3a

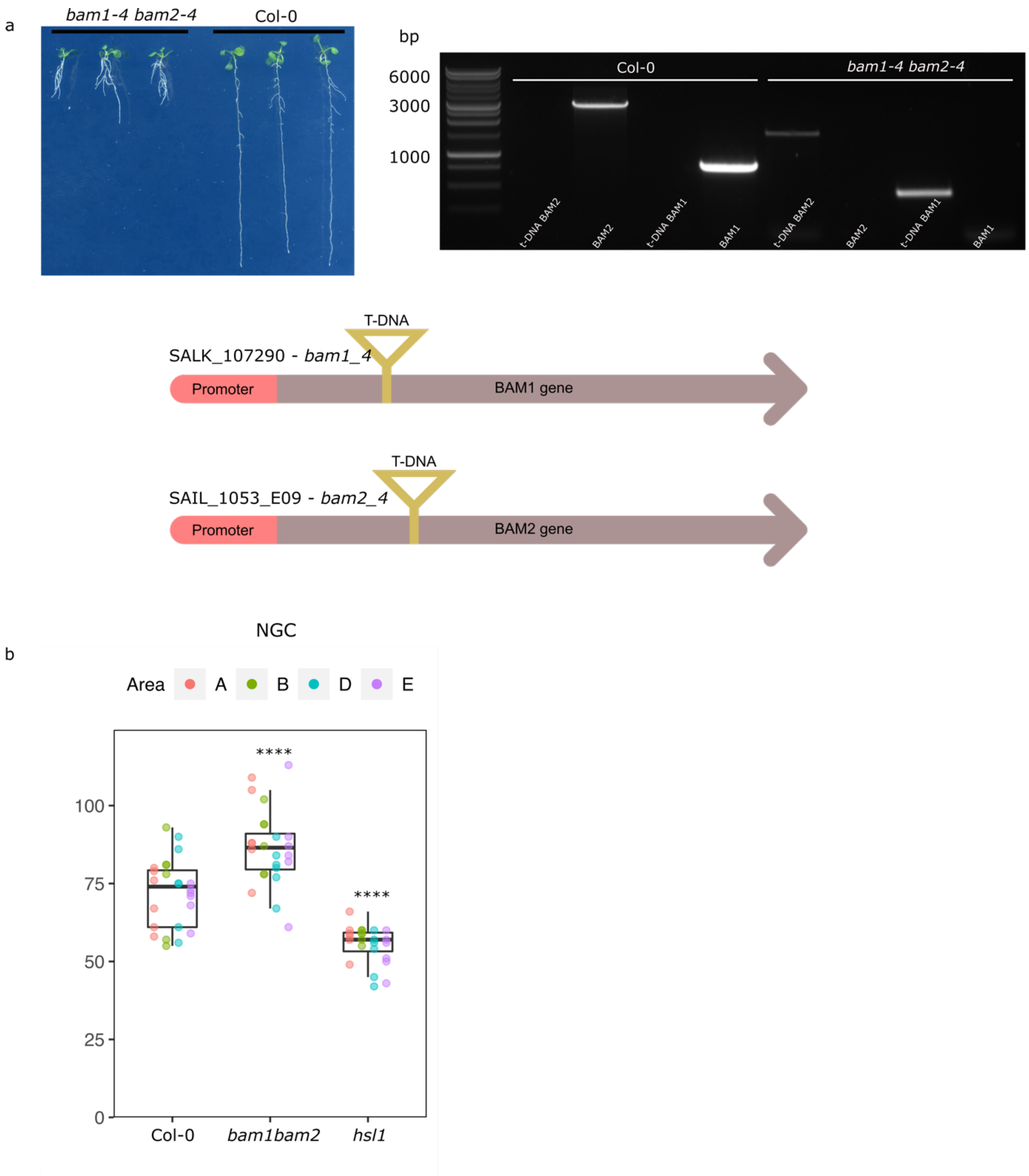

Supplementary Figure 11. Genetic and phenotypic analysis of *bam1-4 bam2-4* mutant. a *bam1bam2* display a short root phenotype. Six-day-old seedlings of *bam1-4bam2-4* double mutant and Col-0 wild type control. Right panel, genotyping analysis of the double mutant *bam1-4bam2-4*. Bottom, gene diagram with the position of the T-DNA insertion in the corresponding *bam1-4* and *bam2-4* alleles of the double mutant. **b** Box plot representing the number of non-guard cells (NGCs) of Col-0 vs *bam1-4bam2-4* and *hsl1-2*. * p < 0.05, ** p < 0.01, *** p<0.001 and **** p<0.0001 by two-sided mixed effect model with a Poisson link function and area as a random factor. The different areas counted per cotyledon are depicted in different colors. n = 2457, 3028, 1822 cells were counted over 6 independent cotyledons for Col-0, *bam1-4 bam2-4* and *hsl1-2*, respectively. The centre line of the box plots represents the median value (50th percentile), while the box contains the 25th to 75th percentiles of the dataset. The black whiskers mark the 5th and 95th percentiles, and values beyond these upper and lower bounds are considered outliers.

a

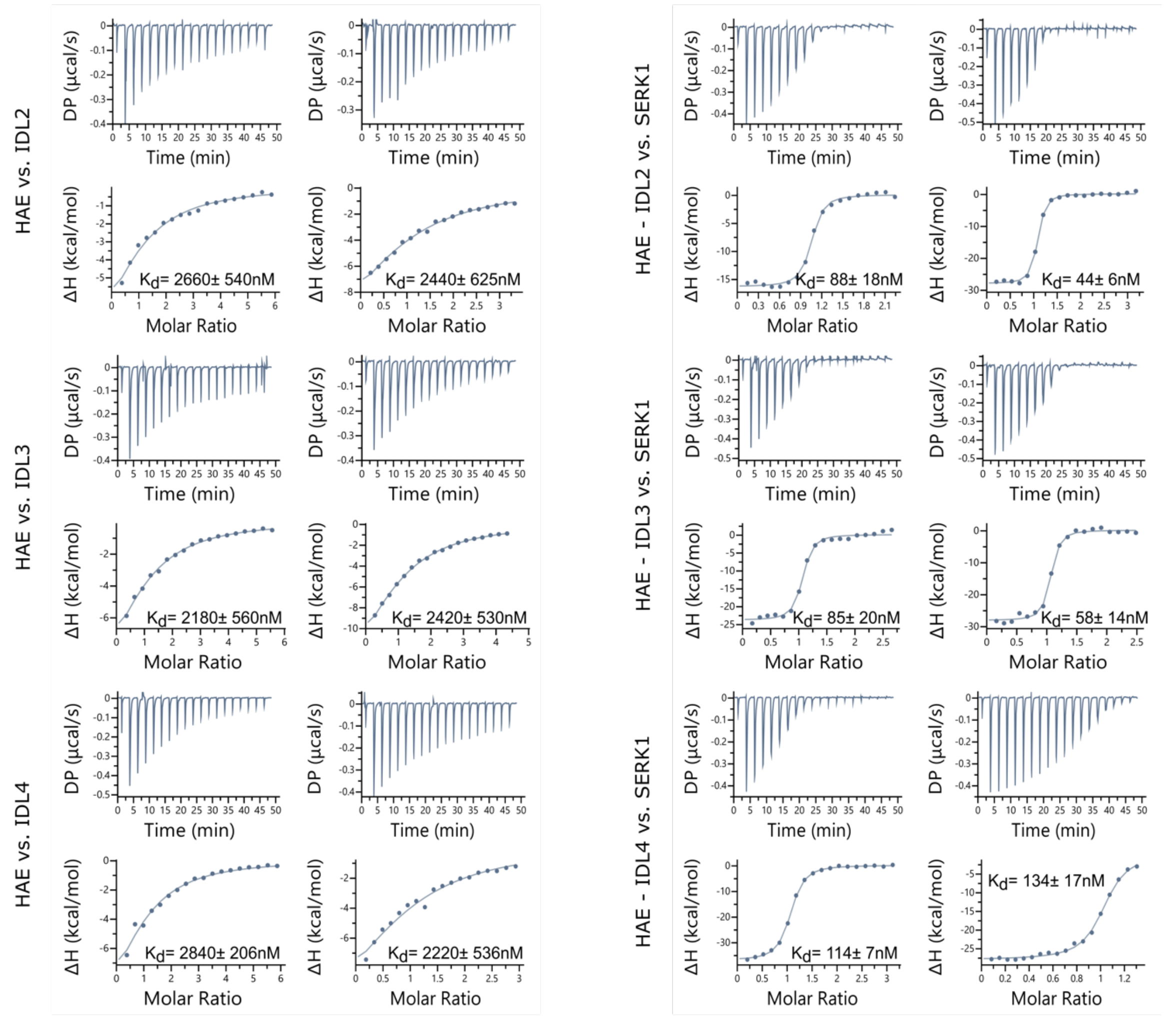

b

Ligand(syringe)	Protein(cell)	$K_{d(nM)}$	ΔH (kcal/mol)	N
IDL2	HAE	2550 ± 155	-14 ± 2	1
IDL3	HAE	2300 ± 170	-17 ± 4	1
IDL4	HAE	2530 ± 440	-13 ± 2	1

Ligand(syringe)	Protein (cell)	$K_{d(nM)}$	ΔH (kcal/mol)	N
SERK1	HAE + IDL2	66 ± 31	-26 ± 1.4	1
SERK1	HAE + IDL3	71.5 ± 19	-26± 3	1
SERK1	HAE + IDL4	124 ± 14	-32± 6	1

c

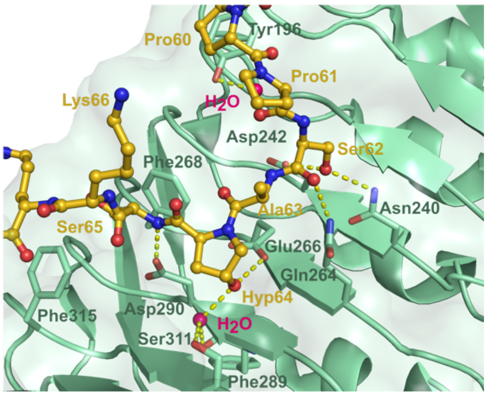

Supplementary Figure 12. The receptor HAESA binds IDA/IDLs with lower affinity than HSL1. **a** ITC thermograms of the independent experiments of HAESA vs IDLs, in the presence and absence of the co-receptor SERK1. **b** ITC summary table. Kd, (dissociation constant) indicates the binding affinity between the two molecules considered (in nanomolar). The N indicates the reaction stoichiometry (n = 1 for a 1:1 interaction). The values indicated in the table are the mean ± SD of at least two independent experiments. **c** Zoom in of the HAESA core region in complex with IDA (PDB: 5IXQ). The two Phe residues in HAESA, F268 and F315, prevent the formation of the extended hydrogen bond network displayed by the two corresponding Tyr (Y261 and Y308) residues in HSL1 (Fig. 2d).

Supplementary Table 1. Crystallographic data reduction and refinement statistics.

PDB ID	Apo_HSL1<br>7ODK	HSL1-IDA-SERK1<br>7ODV	HSL1-IDL1-SERK1<br>7OGO	HSL1-IDL2-SERK1<br>7OGQ	HSL1-IDL3-SERK1<br>7OGZ	HSL1-CLE9-SERK1<br>7OGU
**Data-reduction**						
Space group	*P 1*	*P 21 21 21*	*P 21 21 21*	*P 2 21 21*	*P 21 21 21*	*P 1 21 1*
Wavelength (Å)	*1.000033*	*1.000000*	*0.999990*	1.254716	1.000029	1.000029
Cell dimensions						
a, b, c (Å)	77.54, 84.00, 89.06	97.33, 145.88 169.54	98.74, 146.41, 169.65	86.82, 88.08, 166.37	89.84, 144.50, 168.43	93.96, 169.89, 143.55
α, β, γ (°)	99.18, 113.73, 108.41	90.00, 90.00, 90.00	90.00, 90.00, 90.00	90.00, 90.00, 90.00	90.00, 90.00, 90.00	90.00, 96.74, 90.00
Resolution (Å)	47.08 – 1.83<br>(1.86 -1.83)	110.58 - 2.31<br>(2.35 - 2.31)	169.64 - 2.38<br>(2.42 - 2.38)	46.93 - 2.20<br>(2.25 - 2.20)	48.17 - 2.70<br>(2.77- 2.70)	49.09 - 2.87<br>(2.92 - 2.87)
R_{meas}*	0.059 (0.828)	0.329 (3.606)	0.353 (3.799)	0.123 (2.204)	0.350 (2.153)	0.250 (1.995)
CC(1/2) *(%)**	99.90 (72.10)	99.80 (70.30)	99.80 (66.90)	99.90 (46.50)	99.70 (82.10)	99.60 (54.00)
$I/\sigma I$*	10.3 (1.2)	8.3 (1.2)	9.5 (1.1)	14.5 (1.2)	5.7 (1.3)	7.5 (1.1)
Completeness (%)*	97.4 (96)	100.00 (99.10)	100.00 (100.00)	100.00 (100.00)	99.90 (99.70)	99.60 (92.40)
Redundancy*	3.5 (3.6)	26.0 (26.7)	26.0 (25.70)	12.90 (10.7)	10.3 (10.9)	6.90 (6.7)
Wilson B-factor	28.58	36.90	41.05	42.33	41.16	55.47
**Refinement**						
Resolution (Å)	47.08 – 1.83<br>(1.86 -1.83)	110.58 - 2.31<br>(2.35 - 2.31)	169.64 - 2.38<br>(2.42 - 2.38)	46.93 - 2.20<br>(2.25 - 2.20)	48.17 - 2.70<br>(2.77- 2.70)	49.09 - 2.87<br>(2.92 - 2.87)
No. reflections	158310	106364	99190	65575	60957	101500
$R_{work}/R_{free}$$	0.204 / 0.216	0.255 / 0.275	0.234 / 0.248	0.198 / 0.234	0.353 / 0.371	0.238 / 0.261
No. atoms						
Protein	8907	11835	11794	5936	11603	23236
Glycan	643	624	703	558	428	1147
R.m.s deviations$						
Bond lengths (Å)	0.003	0.004	0.005	0.009	0.005	0.006
Bond angles (º)	1.102	1.250	1.264	1.452	1.366	1.308
Molprobity results						
Ramachandran outliers (%)#	0.00	0.00	0.00	0.00	0.13	0.00
Ramachandran favored (%)#	98.38	98.15	98.07	98.22	98.26	98.65
Molprobity score#	0.87	1.01	1.04	1.01	1.36	1.13

Highest resolution shell is shown in parenthesis.
*As defined in Xia2 /Dials[50]
$As defined in Refmac5[57]
#As defined in Molprobity

Supplementary Table 2. Sequences of the synthetic peptides

Name	Sequence (N->C)
$CLE9^{Hyp112PHyp115P}$	RLVPSGPNPLHN
CLE9	RLV(Hyp)SG(Hyp)NPLHN
$CLE9^{R109PL110I}$	PIVPSG(HYP)NPLHN
$CLE9^{R109P}$	PLVPSG(HYP)NPLHN
$CLE9^{R109PV111P}$	PLPPSG(Hyp)NPLHN
$CLE9^{V111P}$	RLPPSG(Hyp)NPLHN
$CLE9^{R109PL110IV111P}$	PIPPSG(Hyp)NPLHN
$CLE9^{N116S}$	RLV(Hyp)SG(Hyp)SPLHN
CLV3	RTVPSGPDPLHHH
CLE13	RLVPSGPNPLHH
YIDA	YPIPPSA(Hyp)PSKRHN
IDA	PIPPSA(Hyp)SKRHN
$YIDA^{Hyp64P}$	YPIPPSA(Hyp)SKRHN
$YIDA^{K66\ R67\ H68A}$	YPIPPSA(Hyp)SAAAN
YIDL1	YLVPPSG(Hyp)SMRHN
YIDL2	YPVPASG(Hyp)SRKHN
IDL2	PVPASG(Hyp)SRKHN
IDL3	PVPTSG(Hyp)SRKHN
IDL4	PVPASA(Hyp)SRKHN
$IDA^{P58RI59LP60V}$	RLVPSAHypSKRHN

Supplementary Table 3. Primer list

Purpose	Name	Sequence
**Promoters**	pCLE9 Fw	AACAGGTCTCAACCTATCAGAACCTGAGAACTATA
	pCLE9 Rv	AACAGGTCTCTTGTTTGTTTTGGTTTCCAAGAGAGAGCGA
	pHSL1 Fw	AACAGGTCTCAACCTTGTTGCTAATAAGCATTGAGGAGCGTAATGCC
	pHSL1 Rv	AACAGGTCTCTTGTTGTTTCTTCGTCTTCCCCGGTATCTATGACGG
	pIDL4 Fw	AACAGGTCTCAACCTCTTATCTATGACTCGTCATTG
	pIDL4 Rv	AACAGGTCTCTTGTTTTCTATTGTTGAGGATTTTTTGT
	pHAE Fw	AACAGGTCTCAACCTATCTTCAATTGTTTTTTCGTTAATCAC
	pHAE Rv	AACAGGTCTCTTGTTGCTTTGGATTTGTGAATAAAACG
	pHAE2 Fw	AACAGGTCTCAACCTCTTCAATTGTTTTTTCGTTAATCACTCTGC
	pHAE2 Rv	AACAGGTCTCTTGTTTTTTTTGGAAAAGGAATCGTTATTCTTCTTTTTTC
	pIDA Fw	AACAGGTCTCAACCTTTTTCAATTTTGTTATTGCATCTT
	pIDA Rv	AACAGGTCTCTTGTTTTGGTAGTCAATGTTTTTTTTC
**Genes for transgenic lines**	HAE Fw	AACAGGTCTCAGGCTCAACAATGCTTTATTGCTTTATTCTC
	HAE Rv	AACAGGTCTCTCTGAAACGCTGTTCAAGTCTTCCG
	IDA Fw	AACAGGTCTCAGGCTCAATGGCTCCGTGTCGTACG
	IDA Rv	AACAGGTCTCTCTGAATGAGGAAGAGAGTTAACAAAA
**Genotyping**	HSL1_LP	GATTTCTCCTCCGTCACTTCC
	HSL1_RP	GAACGAGTTGTTGACGAGCTC
	BAM1_Fw	CCGGTACTCTTTCCCCAGATGTTTCTCATTTACGTC
	BAM1_Rv	CTTATTGGAAGAGAGATCGAGGAGATTTAGTTTACC
	BAM2_Fw	TATGGTTCGCTTTGGTATTGC
	BAM2_Rv	GTTTGATTAAATGCCATTTTCTGCAAATT
**qRT-PCR**	ACTIN2 Fw	TGCCAATCTACGAGGGTTTC
	ACTIN2 Rv	TTCTCGATGGAAGAGCTGGT
	qIDA Fw	TCGTACGATGATGGTTCTGC
	qIDA Rv	GAATGGGAACGCCTTTAGGT
